# YAP1::TFE3 mediates endothelial‐to‐mesenchymal plasticity in epithelioid hemangioendothelioma

**DOI:** 10.1002/1878-0261.70112

**Published:** 2025-08-17

**Authors:** Ant Murphy, Samuel Hartzler, Paula A. Vargas Carranza, Shyaman Jayasundara, Madison E. Yates, Nimod D. Janson, Bozhi Liu, Annaleigh Benton, Majid Kazemian, Jason A. Hanna

**Affiliations:** ^1^ Department of Biological Sciences Purdue University West Lafayette Indiana USA; ^2^ Purdue University Institute for Cancer Research West Lafayette Indiana USA; ^3^ Department of Biochemistry and Computer Science Purdue University West Lafayette Indiana USA

**Keywords:** EndMT, epithelioid hemangioendothelioma, rare cancer, TFE3, vascular sarcoma, YAP1

## Abstract

The rare vascular sarcoma epithelioid hemangioendothelioma (EHE) is defined by *WWTR1* or *YAP1* gene rearrangements that result in functional fusion proteins. Previous studies have demonstrated the ability of these gene fusions to function as constitutively active TEAD coactivators, while also retaining the ability to drive transcription of canonical CAMTA1 or TFE3 genes, respectively. To better understand the biology underlying EHE, we generated EHE *in vitro* models using endothelial cell lines and found that inducible expression of YAP1::TFE3 (YT) caused a significant change in cellular plasticity. Specifically, YT expression led to endothelial‐to‐mesenchymal transition (EndMT), a process in which endothelial cells lose their highly specialized identity and gain expression of genes typically associated with mesenchymal cells. This plasticity is associated with anoikis resistance and increased migratory phenotypes. Notably, YT drives this phenotypic change independent of TEAD activity but requires dimerization and DNA binding domains encoded by the C‐terminal *TFE3* gene. Overexpression of *TFE3* is insufficient to fully recapitulate the EndMT phenotypes driven by YT; implying that, although dispensable for EndMT, YAP‐TEAD activity provides a meaningful contribution. This work supports a growing body of evidence that YT and *WWTR1‐CAMTA1* driven EHE may have distinct biological mechanisms, underscoring a potentially targetable oncogenic molecular dependency.

## Graphical abstract

The YAP1::TFE3 fusion protein drives endothelial‐to‐mesenchymal transition (EndMT) plasticity, resulting in the loss of endothelial characteristics and gain of mesenchymal‐like properties, including resistance to anoikis, increased migratory capacity, and loss of contact growth inhibition in endothelial cells.

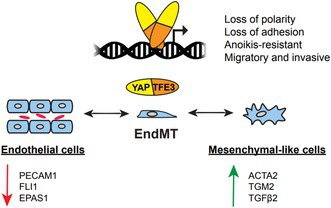

**Abbreviations**
AS: angiosarcoma
bHLH‐LZ: basic helix–loop–helix leucine zipper
co‐IP: co‐immunoprecipitation
DAVID: Database for Annotation, Visualization, and Integrated Discovery
dox: doxycycline
EHE: epithelioid hemangioendothelioma
EndMT: endothelial‐to‐mesenchymal transition
GO: gene ontology
GSEA: gene set enrichment analysis
H&E: hematoxylin and eosin
HUVEC: human umbilical vein endothelial cells
polyHEMA: poly(2‐hydroxylethyl methacrylate)
TBD: TEAD binding domain
TC: TAZ::CAMTA1
YT: YAP1::TFE3

## 1 Introduction

Endothelial‐to‐mesenchymal transition (EndMT) is a process by which endothelial cells lose their quiescent phenotype through loss of expression of endothelial‐specific genes and gain expression of genes associated with mesenchymal cells [1]. EndMT enhances migration, anoikis resistance, and glycolysis to support tumor maintenance and metastatic potential [2, 3, 4]. EndMT occurs during development but is dysregulated in many pathologies including atherosclerosis, vascular malformations, and tumor angiogenesis [5, 6]. However, its role in malignant vascular sarcomas such as angiosarcoma and epithelioid hemangioendothelioma (EHE) is poorly understood.

EHE is an ultra‐rare vascular sarcoma affecting 0.4 cases per million per year. This rarity hinders the study of the mechanisms driving pathogenesis, causing a lack of diagnostic tools and optimized treatments [7, 8, 9]. Patients face a 5‐year survival of 50.7% due to high rates of recurrence and metastasis [8, 10, 11].

EHE is defined by a chromosomal translocation event resulting in functional fusion gene products: WWTR1::CAMTA1, YAP1::TFE3, or the recently discovered case of a WWTR1::TFE3 fusion [12]. TAZ (encoded by *WWTR1*) and YAP1 are paralogous proteins and the major downstream effectors of the Hippo pathway via TEAD transcriptional coactivation [13]. The Hippo pathway is a highly conserved kinase cascade and primary controller of tissue growth, which regulates the cell cycle [14], metabolism, and extracellular matrix remodeling [15]. 10% of all cancers contain mutations in components of the pathway [16] and overexpression of YAP1/TAZ contributes to cancer stem cell proliferation and chemoresistance [15, 17]. Although mutations in YAP1 and TAZ are rare, YAP1/TAZ fusion proteins have been identified in several cancer types [16].

Previous studies have examined the contributions of TEAD transcriptional activity to EHE biology and transformation [18, 19, 20, 21]. Researchers have generated conditional *Wwtr1‐Camta1* transgenic mouse models, which produce tumors that histologically resemble human EHE [19, 22]. Merritt *et al*. [23] found increased TEAD as well as CAMTA1 and TFE3 regulated gene expression for TAZ::CAMTA1 (TC) and YAP1::TFE3 (YT) expressing models, respectively, using NIH3T3 and sarcoma cell models.

TFE3, a basic helix–loop–helix leucine zipper (bHLH‐LZ) MiT/TFE family transcription factor, dimerizes via its bHLH‐LZ domains and primarily binds to CLEAR promoter elements to regulate the expression of genes involved in autophagy and metabolic function [24, 25]. TFE3 genomic fusion events are involved in multiple tumor types [26, 27, 28, 29, 30]; YT‐specific gene rearrangements have been reported in four distinct tumor types: clear cell stromal tumor of the lung [31], PEComas [32, 33], low‐grade fibromyxoid neoplasm [34], and EHE [35]. Clinical evidence suggests distinct clinical and pathological features of YT compared to TC‐EHE tumors, leading to suggestions to reclassify YT‐EHE as a distinct subtype: YAP1::TFE3 fused hemangioendothelioma [36].

To further understand how TC or YT impact the biology of endothelial cells, we transduced endothelial cell lines to inducibly express the EHE fusion proteins. Building on the observation that YT expression induced EndMT, we further investigated the relative contributions of N‐terminal TEAD binding and C‐terminal TFE3 activities of YT in this process.

## 2 Materials and methods

### 2.1 Cell culture

Cells were obtained from the following sources: Pooled primary HUVEC (PCS‐100‐013), HMEC‐1 (CRL‐3243, RRID: CVCL_0307), MS1 (CRL‐2279, RRID: CVCL_6502) from American Type Culture Collection (ATCC, Manassas, VA, USA), and HEK‐293T (RRID: CVCL_0063, M. Kazemian, Purdue University). All cells undergo mycoplasma testing at least three times annually, and the cell lines were verified by STR profiling by IDEXX Cell Check STR profiles. Cells were treated with the indicated concentration of K‐975 (HY138565, MedChemExpress, Monmouth Junction, NJ, USA), terfenadine (HYB1193100MG, MedChemExpress), doxycycline hyclate (446061000m Thermo Fisher Scientific, Waltham, MA, USA), or vehicle controls. Cell viability was determined using the Cell Titer Glo Assay (G7570, Promega, Madison, WI, USA), and apoptosis was determined using the Caspase 3/7 Glo Assay (G8090, Promega). HMEC‐1 cells were maintained in MCDB131 (15100CV Corning, NY, USA) supplemented with 10 ng·mL^−1^ EGF (23 626, R&D Systems, Minneapolis, MN, USA), 1 μg·mL^−1^ hydrocortisone (0219456901, MP Biomedicals, Irvine, CA, USA), 1× Glutamax (35050, Gibco/Thermo Fisher Scientific, Waltham, MA, USA), and 10% FBS (SH3091003, Hyclone, Logan, UT, USA). HUVECs were maintained in EBM‐2 Basal Medium supplemented with EGM‐2 SingleQuots (CC‐3162, Lonza, Walkersville, MD, USA). The MS1 and HEK‐293T cells were maintained in DMEM (SH30243, Hyclone) with 10% FBS (SH30910.03, Hyclone), 1× antibiotic‐antimycotic penicillin, streptomycin, and amphotericin B (PSA) (A5955, Sigma‐Aldrich, St. Louis, MO, USA) and incubated at 37 °C in 5% CO_2_.

Low adherent plates were prepared using poly(2‐hydroxylethyl methacrylate) (polyHEMA) (P3932, Millipore, Burlington, MA, USA) as a plate coating. Focus assays were performed by seeding 1.5 × 10^5^ cells in a six‐well dish. After 72 h of doxycycline treatment, cells were then crystal violet stained, imaged, and quantified using ImageJ. Three independent fields per sample were used for quantification of images. Scratch assays were performed by seeding doxycycline‐pretreated cells at 2.0 × 10^5^ cells per well and incubating for 24 h before scratching the surface of the culture dish with a p200 micropipette tip. Scratches were imaged at hour 0 and hour 16 post scratch. Wound size area was measured using ImageJ, and at least three independent fields per sample were used for quantification of images.

### 2.2 RNA and gene expression

Total RNA from cells was prepared using the *Quick*‐RNA Miniprep Kit (R1054, Zymo, Irvine, CA, USA) or tissue using the *Quick*‐RNA Miniprep Plus Kit (R1057, Zymo) according to the manufacturer's instructions. cDNA was generated using High‐Capacity RNA to cDNA kit (4387406, Applied Biosystems, Waltham, MA, USA). Relative expression by qRT‐PCR using primers detailed in Table S1 and quantified using the delta–delta CT method normalized to *TBP*. RNA‐seq was performed on RNA from MS1 cells expressing YT or RFP control by Novogene on an Illumina NovaSeq platform and analyzed as described previously [37]. Differentially expressed genes were analyzed for gene ontology enrichment using the Database for Annotation, Visualization, and Integrated Discovery (DAVID) [38]. Functional classification of terms or pathways were analyzed based on biological process gene ontology terms (BP_FAT) with the exclusion of redundant terms. Gene Set Enrichment Analysis (GSEA) [39, 40] conducted with previously defined gene sets including: 93‐gene EHE set [20], the Hallmark Epithelial Mesenchymal Transition gene set, and the Descartes Organogenesis Endothelial Cells gene set. Raw paired‐end sequencing data were processed, and gene expression was quantified using rsem [41] (version 1.3.1) with default parameters, with bowtie2 [42] as the aligner. The required bowtie2 reference was prepared using “rsem‐prepare‐reference” for the mouse (GRCm38) transcriptome with RefSeq genes, downloaded from UCSC. Differentially expressed gene (DEG) analysis was performed using deseq2 [43].

### 2.3 Molecular cloning and viral transduction

2X FLAG‐tagged doxycycline inducible plasmids were cloned from YAP1, TFE3, YAP1::TFE3, and TAZ::CAMTA1 expressing plasmids provided by Munir Tanas [18, 23] using primers detailed in Table S2. The YAP1::TFE3 plasmid fuses exon 1 of *YAP1* to exon 4 of *TFE3* as identified in an EHE patient [35]. The HA‐tagged TEAD1 cDNA was cloned from the HA‐TEAD1 plasmid (Addgene #229411) provided by Christopher Vakoc [44]. Using standard gateway or Gibson Assembly cloning (into AgeI/NheI sites), the cDNAs were cloned into pCW57.1 (Addgene #41393 provided by David Root) for MS1 and HUVEC transductions or pINDUCER20 (Addgene #44012, provided by Stephen Elledge [45]) for the HMEC‐1 cells. Lentivirus was generated and packaged in HEK‐293Ts as previously described [46, 47]. Forty‐eight hours post transduction, cells were selected and maintained in 2 μg·mL^−1^ puromycin (BP2956100, Fisher Bioreagents, Waltham, MA, USA) or 500 μg·mL^−1^ G418 (BP6735, Fisher Bioreagents). YT^S94A^ and TFE3^S47A^ were generated with site‐directed mutagenesis using the QuickChange II mutagenesis kit and primers in Table S2. YT^Δdd^ was generated by first removing the bHLH and LZ domains of TFE3 using endogenous BglII and EcoRI digestion, followed by ligating annealed oligos detailed in Table S2 to generate pDONR221‐2xFLAG‐TFE3^Δdd^. The Δdd fragment was then digested out of the pDONR221‐2xFLAG‐TFE3^Δdd^ plasmid with BglII and EcoRV and ligated into previously digested pDONR221‐YT to generate pDONR221‐2XFLAG‐YT^Δdd^.

### 2.4 Immunoblots and co‐immunoprecipitations

Protein lysates were prepared in RIPA buffer with 1x Halt Protease Inhibitor (78430, Thermo Fisher Scientific) as described [48]. The BCA Protein Assay Kit (23225, Pierce, Rockford, IL, USA) was used to determine protein concentrations. Lysates were resolved by SDS/PAGE using NuPAGE 4–12% Bis‐Tris Gel (NP0321BOX, Invitrogen/Thermo Fisher Scientific) and transferred to Immobilon‐P Transfer Membrane (IPVH00005, Merck Millipore). Blots were probed overnight at 4 °C with primary antibodies (detailed in Table S3). Membranes were then washed and probed with secondary antibodies conjugated with HRP. Antibody‐bound protein was visualized using chemiluminescence Luminol reagent (SC‐2048, Santa Cruz Biotechnology, Dallas, TX, USA) and film (45‐001‐508 Amersham Hyperfilm ECL Film, Cytiva, Marlborough, MA, USA) or Azure c600 imager (Azure Biosystems, Dublin, CA, USA). Cell lysates were prepared in Tris EDTA buffer with 1× Halt Protease Inhibitor Cocktail. For pulldowns, Pierce Protein A/G Magnetic Agarose beads (78609, ThermoFisher Scientific) were coated with anti‐TEAD antibody (132955, Cell Signaling Technology) before incubation with lysate for 10 min at RT. Lysate was boiled to elute from the beads, and the resultant protein solution was used for immunoblotting as described above.

### 2.5 Immunofluorescence and immunohistochemistry

Cells were fixed for 10 min using 4% paraformaldehyde (043368‐9M, Thermo Scientific), permeabilized using Triton X‐100 (BP151‐500 Fisher Scientific), incubated overnight at 4 °C with anti‐FLAG antibody (14793, Cell Signaling Technologies), and subsequently incubated with secondary antibody (A‐21245, goat anti‐rabbit Alexa Fluor Plus 647, Thermo Fisher Scientific) for 1 h at RT. DAPI (62247, Thermo Fisher Scientific) was incubated for 10 min prior to mounting. H&Es and ⍺‐SMA staining were conducted by the Purdue Histology Research Laboratory following standard methods (detailed in Table S3).

### 2.6 Tumor allograft studies

All mouse studies were reviewed and approved by the IACUC at Purdue University (PACUC protocol #1908001941). Mice were fed and watered *ad libitum* in a facility maintained at ambient temperature and humidity with 12‐h light/dark cycles. 2 × 10^6^ RFP‐MS1 or YT‐MS1 cells resuspended in Matrigel were subcutaneously injected into the right flank of 8‐week‐old NRG (Strain 007799, NOD.Cg‐*Rag1tm1Mom Il2rgtm1Wjl*/SzJ, Jackson Laboratory, Bar Harbor, ME, USA) mice (*n* = 4 each group, both genders, randomized). Mice were then given 1 mg·mL^−1^ doxycycline in 2% sucrose water, and tumors were measured weekly. The cohort was euthanized when the first tumor volume of 2 cm^3^ was reached (130 days after injection).

### 2.7 Statistics

Statistical analyses were performed using prism Version 9 (Graph Pad Software, Inc., San Diego, CA, USA). All experiments were performed in three independent biological replicates, with each experiment including at least three technical replicates. All results are expressed as the mean ± SD from one representative biological replicate unless stated otherwise. Pairwise comparisons were performed with a two‐tailed, unpaired Student's *t*‐test. Significance cutoff at *P* values < 0.05.

## 3 Results

### 3.1 *In vitro* endothelial EHE cell models have unrestrained Hippo activity

**Fig. 1:**
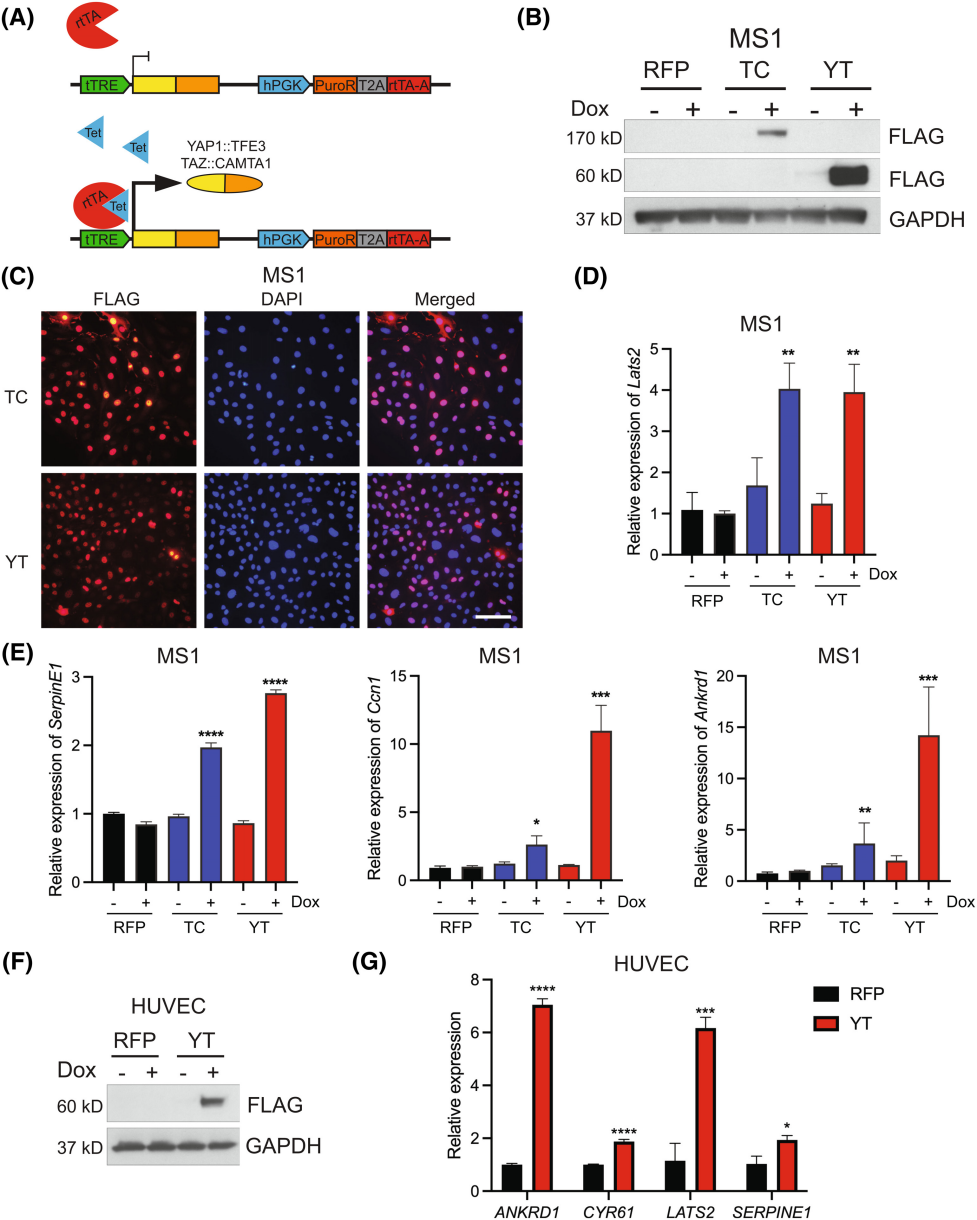
Generation of endothelial EHE cell models. (A) Graphic of doxycycline (dox) inducible expression of YAP1::TFE3 (YT) and TAZ::CAMTA1 (TC) pCW57.1 lentiviral system. (B) Immunoblot analysis of MS1 cell lysates post 72 h of ±1 μg·mL^−1^ dox treatment and probed with anti‐FLAG or GAPDH as indicated (*n* = 3). (C) Immunofluorescent staining of indicated MS1 cells probed using anti‐FLAG antibody in dox treated cells as in (B), scale bar 100 μm (*n* = 3). (D, E) Relative expression of indicated genes by qRT‐PCR in MS1 cells as in (B) (*n* = 3). (F) Immunoblot of lysates from stably transduced HUVEC cells treated ±1 μg·mL^−1^ dox for 72 h and probed with anti‐FLAG or GAPDH (*n* = 3). (G) qRT‐PCR analysis of RNA from HUVEC cells as in (F). Error bars indicate standard deviation, significance *P < 0.05, **P < 0.01, ***P < 0.001, ****P < 0.0001 determined by Student's two‐tailed *t*‐test (*n* = 3).

To determine the transformative potential of YT and TC in endothelial cells, we stably transduced the immortalized murine endothelial cell line (MS1) [49] and human immortalized microvascular endothelial cells (HMEC‐1) [50] with FLAG‐tagged TC or YT under a doxycycline (dox)‐inducible promoter (Fig. 1A). HMEC‐1 and MS1 fusion protein expression and predominant nuclear localization were observed in a dox‐dependent manner even when cells reached confluency (Fig. 1B,C, Fig. S1A,B). Hippo pathway‐related genes were transcriptionally upregulated as expected (Fig. 1D,E, Fig. S1C). There were some differences where *SERPINE1* was not increased in the HMEC‐1 cells, likely due to the high baseline in the cells. Additionally, *ANKRD1* was not upregulated by TC in the HMEC‐1 cells.

We also attempted to generate primary human umbilical vein endothelial cells (HUVECs) with the dox‐inducible fusion proteins. We were unable to generate HUVEC cells that maintain high TC expression, consistent with recent findings indicating that TC expression in endothelial cells induces hypertranscription stress and cellular senescence [51]. Previous studies also indicated TC expression is insufficient to transform HUVEC cells [18]. Nonetheless, we observed high YT expression, nuclear localization, and Hippo pathway gene upregulation in YT‐HUVEC cells in a dox‐dependent manner (Fig. 1F,G, Fig. S1D). Due to the overall lower expression of TC and observations that additional genomic alterations enhance TC‐mediated transformation [52], we focused on the less‐studied YT fusion protein in endothelial cell tumorigenesis.

**Fig. 2:**
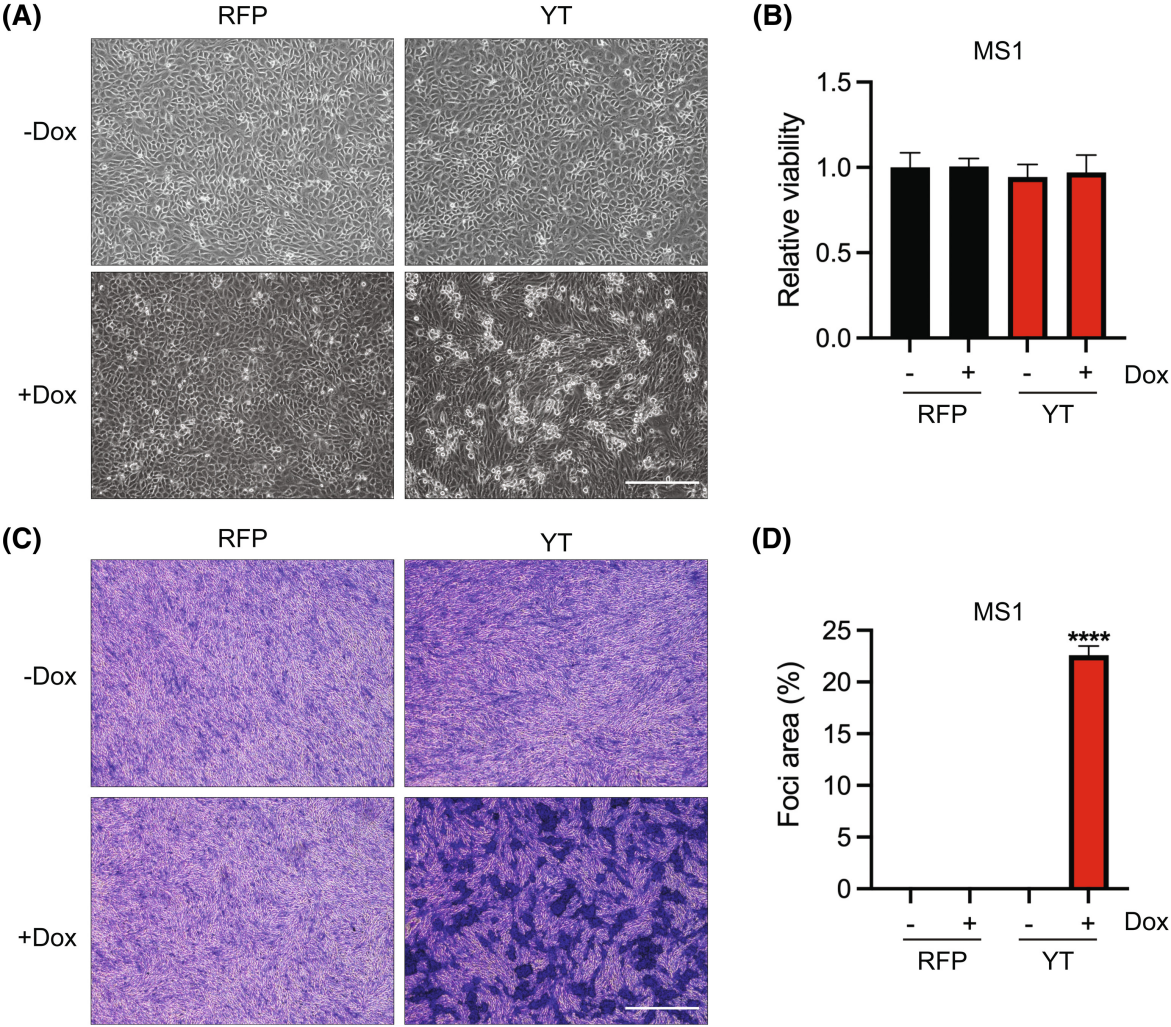
Expression of YAP1::TFE3 drives morphologic alterations and transformation in endothelial cells. (A) Brightfield images of MS1 EHE cells 72 h post ±1 μg·mL^−1^ doxycycline (dox) treatment, scale bar 300 μm (*n* = 3). (B) Relative cell viability of YT‐MS1 cells relative to RFP control with 72 h dox based on Cell Titer Glo (*n* = 3). (C) Representative images of crystal violet stained focus assays and (D) percentage foci area quantification. Scale bar 750 μm (*n* = 3). Error bars indicate standard deviation, significance ****P < 0.0001 determined by Student's two‐tailed *t*‐test.

We observed a distinct change in morphology specific to the YT‐expressing endothelial cells wherein they lost their typical cobble‐stone‐like appearance, becoming elongated and irregularly shaped (Fig. 2A, Fig. S2A). There was no increase in cell viability upon YT expression (Fig. 2B, Fig. S2B). However, YT‐MS1 cells grown beyond 100% confluence continued to proliferate and formed three‐dimensional foci, suggesting a loss of contact‐mediated growth inhibition (Fig. 2C,D). Thus, YT expression results in a loss of typical endothelial morphology and growth suppression by contact inhibition.

### 3.2 YT alters transcriptional program

**Fig. 3:**
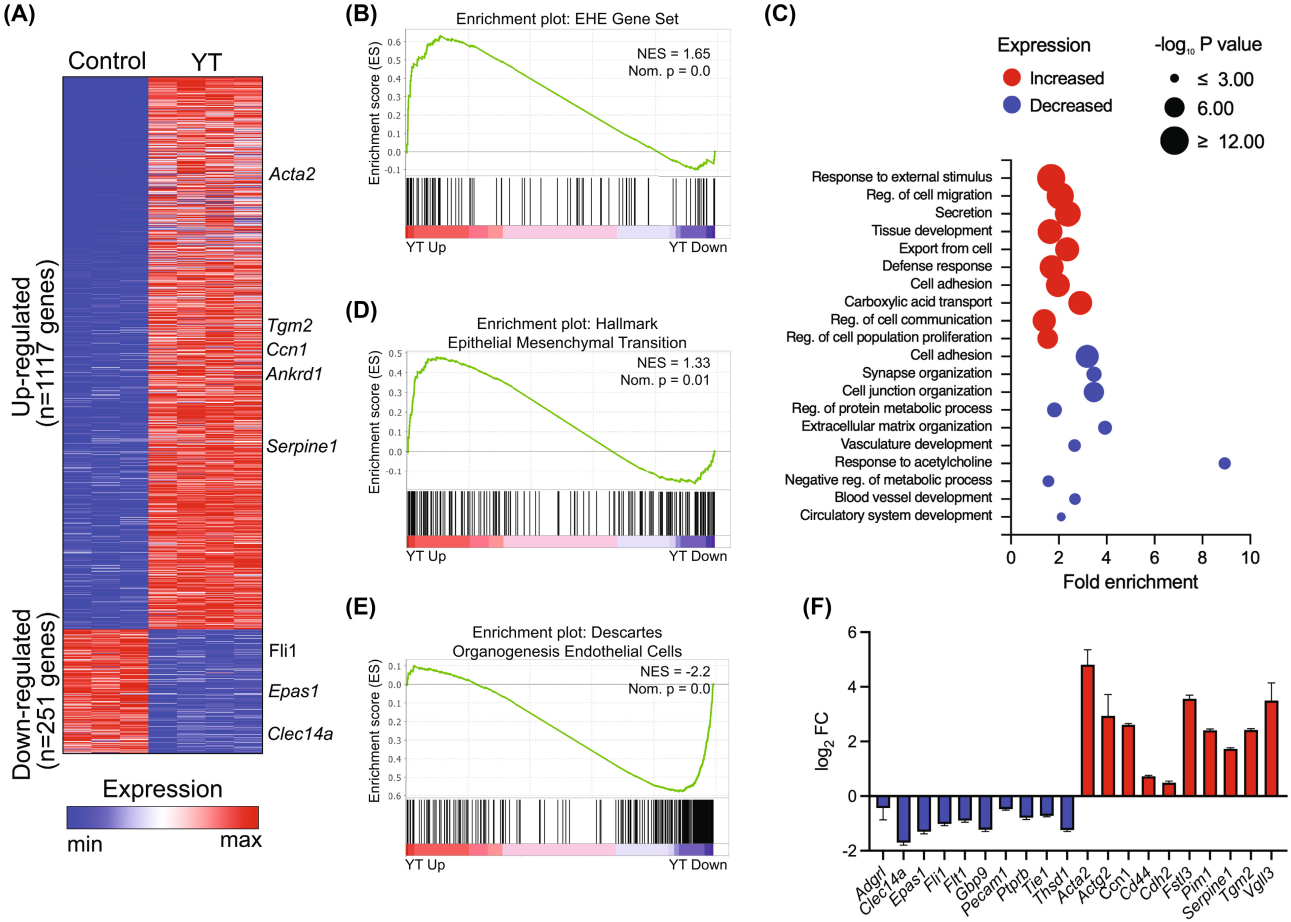
Transcriptional changes in YT‐expressing MS1 cells reveal EndMT plasticity. (A) Heatmap showing the expression of 1117 upregulated and 251 downregulated genes in YT‐expressing MS1 cells compared to RFP control cells (FC > 1 and adjusted *P*‐value < 0.05) (B) Gene Set Enrichment Analysis (GSEA) comparing YT‐MS1 cells to control RFP‐MS1 cells with the human EHE‐specific gene signature. Normalized enrichment score (NES) and nominal *P* values are shown. (C) Gene Ontology (GO) analyses of upregulated (red, top panel) and downregulated (blue, bottom panel) genes in YT‐MS1 cells. (D) GSEA of YT‐MS1 cells against the Hallmark of EMT gene set or (E) Descartes Organogenesis Endothelial Cells gene set. Normalized enrichment score and nominal *P* values are shown. (F) Log_2_ fold change in the expression of selected downregulated endothelial (blue) and upregulated mesenchymal (red) genes in YT‐expressing MS1 cells compared to RFP‐expressing control cells. Error bars represent the standard error of the log_2_ fold change.

To better understand the transcriptional changes underlying the observed phenotypes, we conducted RNA‐sequencing comparing control RFP‐MS1 cells with YT‐MS1 cells (Fig. S3). We identified 1117 upregulated and 251 downregulated genes in YT‐MS1 cells (Fig. 3A). To assess whether YT expression recapitulates EHE patient tumors' transcriptional profiles, we performed GSEA with a 93‐gene EHE‐specific signature [19] and observed a strong enrichment of upregulated genes in YT‐MS1 cells, indicating that they transcriptionally resemble EHE tumors (Fig. 3B).

Gene Ontology (GO) analysis of the 251 downregulated genes identified association with pathways related to cell adhesion and basic endothelial functions such as blood vessel development and ECM organization (Fig. 3C, Table S4). GO analysis revealed that upregulated genes in YT‐expressing MS1 cells were related to cell migration, secretion, and cell adhesion (Fig. 3C, Table S5). Adhesion genes were involved in both up‐ and downregulated gene lists. However, upon further analysis, the genes that are downregulated are more involved in structural or static cell–cell adhesion such as *Alcam*, *Cldn34c1*, several genes of the protocadherin superfamily (*Pcdhb21*, *Pcdhga8*, *Pcdhgc5*, and *Pcdhgc4*), and *Dchs1*, which is involved in cell polarity and adhesion. Whereas adhesion genes that are increased in expression with YT are associated with more cell migration and dynamic/focal adhesions including such genes as *Axl*, *Tln2*, integrins, and signaling molecules associated with migration (*Pdgfra*, *Tgfb2*, and *Ret*). Consistent with this finding, genes upregulated in YT‐expressing cells were highly enriched in the Hallmark gene set for EMT by GSEA (Fig. 3D), while genes downregulated in YT‐MS1 cells were associated with endothelial cell transcriptional signatures from the “Descartes” endothelial single‐cell gene set [53], indicating the loss of endothelial gene expression (Fig. 3E). Indeed, the RNA‐seq data indicate a loss of canonical endothelial genes and an increase in mesenchymal‐associated gene expression (Fig. 3F). These data demonstrate that YT expression drives transcriptomic changes consistent with a promigratory EndMT transcriptional program and a similar gene expression profile observed in EHE patient samples.

### 3.3 YAP1::TFE3 drives EndMT plasticity

**Fig. 4:**
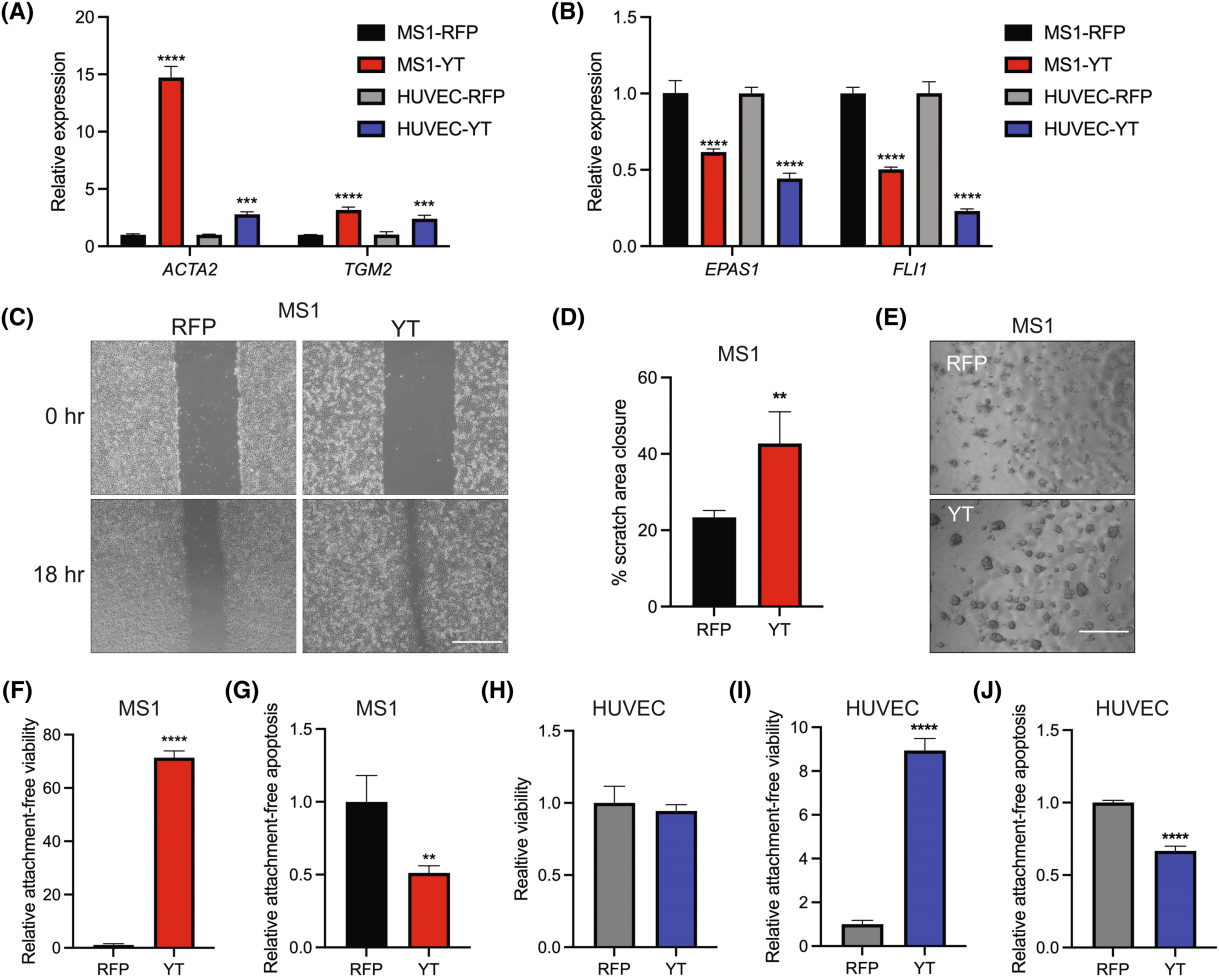
YAP1::TFE3 drives EndMT in endothelial cells. (A) Relative expression of mesenchymal genes, *Acta2* and *Tgm2*, and (B) endothelial *Epas1* and *Fli1* by qRT‐PCR in MS1 or HUVEC cells expressing RFP control or YT treated with 1 μg·mL^−1^ doxycycline (dox) for 72 h (*n* = 3). (C) Representative scratch wound healing images of dox‐treated cells at time 0 and 18 h after scratching, scale bar 750 μm. (D) Quantification of scratch area closure from (C) (*n* = 3). (E) Representative images of indicated YT‐MS1 or RFP control cells cultured in 1 μg·mL^−1^ dox and low‐attachment conditions for 72 h, scale bar 750 μm. (F) Relative viability by Cell Titer Glo and (G) relative Caspase activity by Caspase 3/7 Glo assays of suspension‐cultured YT‐MS1 cells normalized to RFP control cells grown in adherent conditions (*n* = 3). (H) Relative cell viability of YT‐HUVEC or control RFP cells grown in 2D attached conditions or (I) low‐attachment conditions by Cell Titer Glo in cells treated with 1 μg·mL^−1^ dox (*n* = 3). (J) Relative apoptosis activity of HUVEC cells as in (I) (*n* = 3). Error bars indicate standard deviation, significance **P < 0.01, ***P < 0.001, ****P < 0.0001 determined by Student's two‐tailed *t*‐test.

Using qRT‐PCR, we found a significant increase in expression of EndMT gene markers *Acta2* and *Tgm2* and a decrease in endothelial genes *Fli1* and *Epas1* in YT‐MS1 and YT‐HUVEC cells (Fig. 4A,B). This is consistent with the transcriptional changes observed in YT‐MS1 cells by RNA‐seq (Fig. 3A). Immunoblot analysis also demonstrates an increase in ⍺‐SMA protein in YT‐MS1 cells (Fig. S4A). Although a subtle but significant reduction in *Pecam1* expression is observed in the YT‐MS1 cells (Fig. 3F), canonical endothelial genes *PECAM1* and *CDH5* are significantly decreased in the human endothelial cells with YT expression (Fig. S4B). EndMT is associated with promigratory effects on endothelial cells [2], which are typically highly attached and immobile. YT‐MS1 cells were more migratory in a standard scratch healing assay (Fig. 4C,D).

As GO analysis indicated transcriptional changes associated with cell attachment (Fig. 3C), we next determined the ability of YT to promote survival in attachment‐free conditions. YT‐MS1 cells exhibited enhanced survival in low adherent conditions and significantly reduced Caspase 3/7 activity compared with RFP control cells, indicating a decrease in anoikis or apoptosis from loss of adherence (Fig. 4E–G). These differences in attachment‐free viability and apoptosis were recapitulated in YT‐HUVEC cells (Fig. 4H–J). Thus, YT expression causes both transcriptional and phenotypic changes consistent with EndMT.

To determine whether forced expression of YAP1, TFE3, or TEAD1 was sufficient to phenocopy YT effects, we also generated stable MS1 cells with dox‐inducible expression of these wild‐type proteins (Fig. S4C). TEAD1 forced expression did not lead to any phenotypes associated with EndMT or transformation (Fig. S4C–H). YAP1 and TFE3 do increase foci formation when cells are grown past confluence. However, the foci area is not to the extent of YT despite similar expression levels (Fig. S4C,D). Additionally, mesenchymal transcriptional changes were not observed for increased *Acta2* and *Tgm2*. Notably, endothelial *Epas1* and *Fli1* were decreased with YAP1 expression. Therefore, overexpression of YAP1, TFE3, or TEAD1 is unable to fully promote EndMT phenotypes to the extent driven by YT.

### 3.4 TEAD activation is not necessary for YAP1::TFE3 driven EndMT

**Fig. 5:**
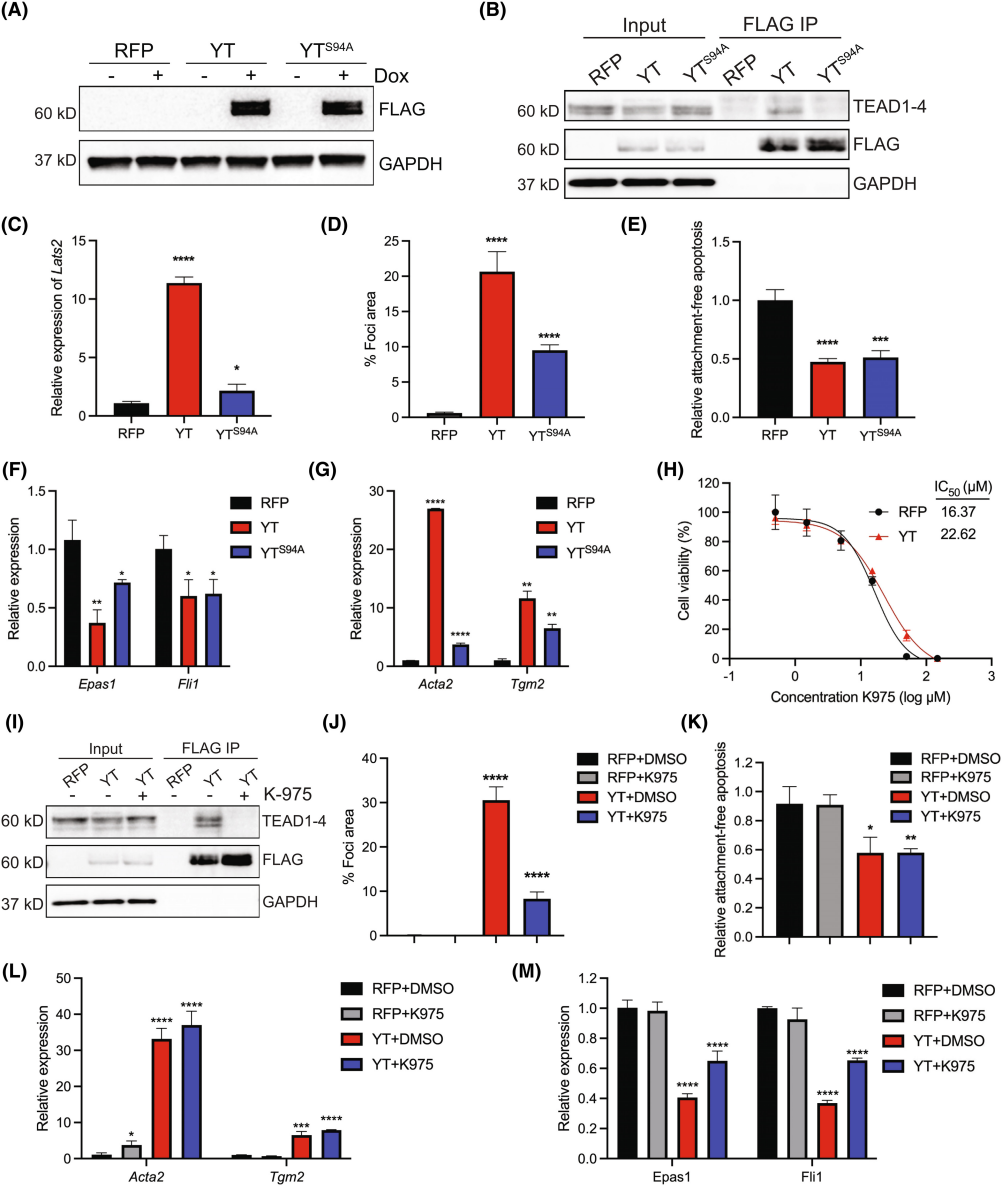
Constitutive TEAD transcription is dispensable for YT‐driven EndMT plasticity in MS1 cells. (A) Immunoblot analysis of inducible YT and YT^S94A^ in MS1 cells treated ±1 μg·mL^−1^ doxycycline (dox) for 72 h (*n* = 3). (B) Immunoblot of indicated input lysates or FLAG co‐IPs in RFP‐MS1, YT‐MS1, or YT^S94A^‐MS1 cells treated with dox (1 μg·mL^−1^) (*n* = 3). (C) Relative expression of *Lats2* by qRT‐PCR (*n* = 3). (D) Focus formation quantification of RFP‐MS1 (black), YT‐MS1 (red), or YT^S94A^‐MS1 (blue) cells treated with dox (1 μg·mL^−1^) (*n* = 3). (E) Relative caspase activity of suspension‐cultured MS1 cells normalized to RFP control cells grown in adherent conditions based on Caspase 3/7 Glo assay (*n* = 3). (F) Relative expression of indicated endothelial or (G) mesenchymal genes by qRT‐PCR in RFP‐MS1, YT‐MS1, or YT^S94A^‐MS1 cells treated with dox (1 μg·mL^−1^) (*n* = 3). (H) Cell viability curve for RFP‐MS1 or YT‐MS1 cells, 72 h after treatment with indicated concentrations of TEAD‐YAP1 interaction inhibitor, K‐975, and dox (1 μg·mL^−1^) (*n* = 3). (I) Immunoblot analysis of input lysate or FLAG co‐IPs in indicated MS1 cells treated ± K‐975 (1 μm) (*n* = 3). (J) Focus assay quantification of indicated MS1 cells treated with dox and K‐975 (1 μm) (*n* = 3). (K) Relative caspase activity of suspension‐cultured MS1 cells normalized to RFP control cells grown in adherent conditions and treated with dox and K‐975 (*n* = 3). (L) Relative expression of mesenchymal or (M) endothelial genes by qRT‐PCR in indicated MS1 cells (*n* = 3). Error bars indicate standard deviation; significance *P < 0.05, **P < 0.01, ***P < 0.001, ****P < 0.0001 determined by Student's two‐tailed *t*‐test.

Since EHE is thought to be driven through constitutive TEAD activity [18, 19, 20, 21], we sought to determine if the ability of YT to bind the TEAD family of transcription factors is necessary for the observed EndMT phenotypes. We generated a mutant version of YT with a mutation of YAP1‐S94 that is vital for YAP1 binding to TEADs [13]. The expression of this variant was similar to YT upon dox induction in the MS1 cells (Fig. 5A). Indeed, we observe less TEAD binding to YT^S94A^ by co‐immunoprecipitation (co‐IP) experiments pulling down cell lysate with anti‐FLAG antibody and probing immunoblots using an anti‐TEAD antibody (Fig. 5B). A reduction in TEAD transcriptional activity in YT^S94A^ compared with YT was observed by qRT‐PCR of the TEAD‐regulated gene, *Lats2* (Fig. 5C). YT^S94A^ cells maintain the ability to form 3D foci, but foci size is reduced compared with YT cells (Fig. 5D, Fig. S5A). MS1 cells expressing YT^S94A^ also maintain resistance to anoikis and are able to survive as clusters in suspension, similar to YT cells (Fig. 5E). Finally, YT^S94A^ expression significantly reduced the expression of endothelial *Fli1* and *Epas1* and increased the expression of mesenchymal *Acta2* and *Tgm2*, consistent with EndMT (Fig. 5F,G). Similar findings were observed with YT^S94A^ expression in HUVEC cells, where YT^S94A^ also maintained resistance to anoikis in suspension conditions (Fig. S5B,C). Additionally, EndMT transcriptional changes were maintained although *ACTA2* increases were more subtle (Fig. S5D).

To further these findings, we utilized pharmacological inhibition of TEADs. K‐975 is a potent allosteric inhibitor that covalently binds in the palmitate binding pocket of TEADs to inhibit YAP/TAZ‐TEAD interactions and transcriptional activity of canonical Hippo pathway target genes [54]. YT‐MS1 cells exhibit only a slightly higher IC_50_ for K‐975 compared with control (Fig. 5H). This is an expected result considering YT‐MS1 cells do not have an increased viability compared with control cells. To assess the efficacy of K‐975 in inhibiting TEAD interactions with YT, we conducted co‐IP experiments. Treatment with 1 μm K‐975 resulted in reduced TEAD protein co‐IP with FLAG compared with the DMSO‐treated control (Fig. 5I). K‐975‐treated YT‐MS1 cells are still able to form 3D foci, albeit less than control YT‐MS1 cells (Fig. 5J, Fig. S5E). K‐975 does not cause YT‐MS1 cells to become sensitive to anoikis and was also not sufficient to block differential expression of EndMT genes (Fig. 5K–M). Finally, K‐975 treatment in YT‐HUVEC cells similarly maintains resistance to anoikis and EndMT transcriptional alterations (Fig. S5F,G). Altogether, these data demonstrate the ability of YT to constitutively activate TEAD is not required for anoikis resistance or loss of contact‐mediated growth inhibition. This suggests TEAD is not necessary for EndMT plasticity but meaningfully contributes to growth phenotypes as expected. Therefore, we interrogated the altered TFE3 transcriptional activity of YT in these processes.

### 3.5 TFE3 dimerization and DNA‐binding domains are required for YT‐driven EndMT plasticity

**Fig. 6:**
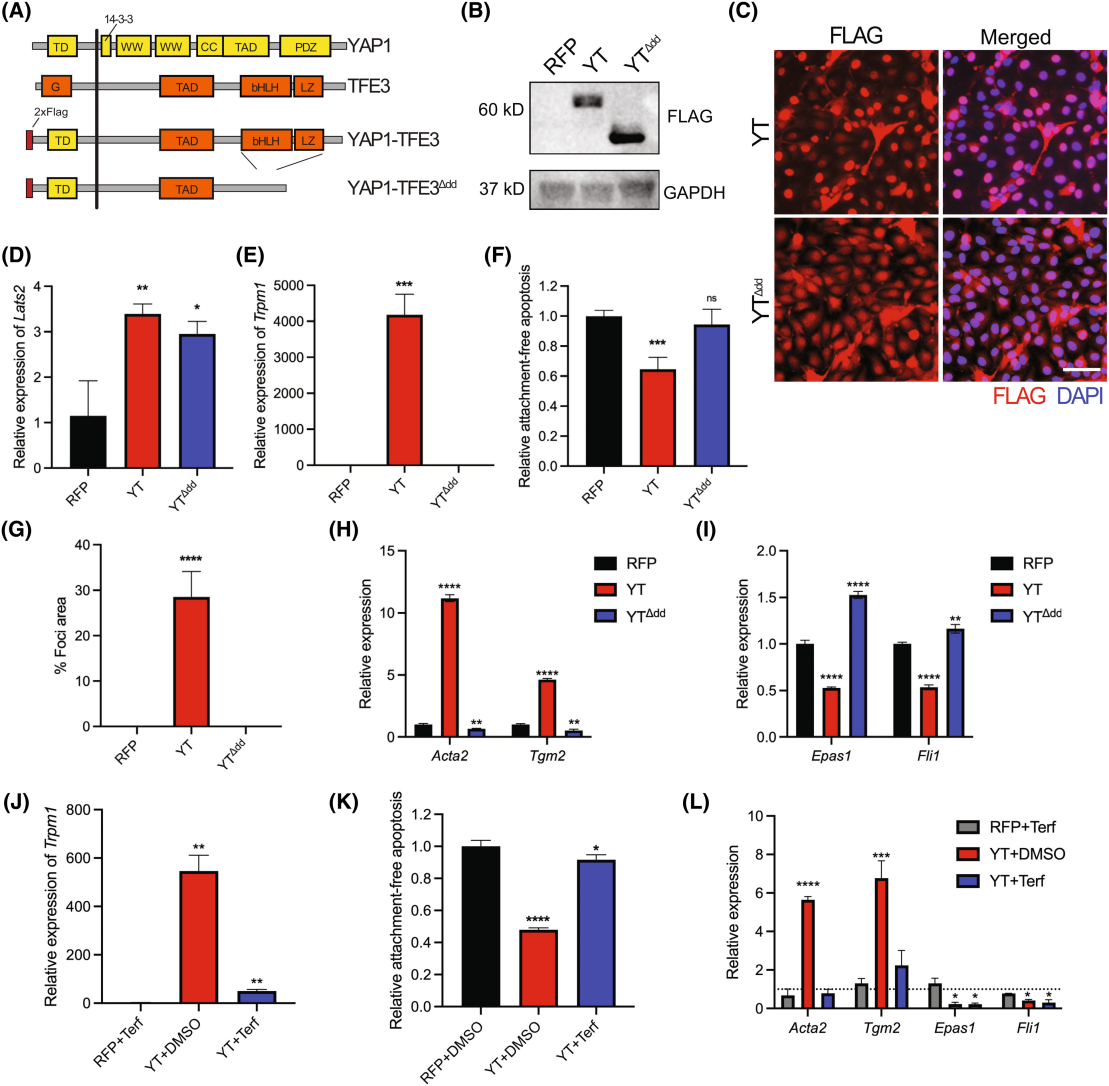
YT‐driven EndMT plasticity is dependent on basic‐helix–loop–helix leucine zipper domains donated by TFE3 in MS1 cells. (A) Graphic showing a map of YT domains and the deletion of the bHLH/LZ domains in the YT^Δdd^ mutant. (B) Immunoblot analysis of MS1 cell lysates from cells stably transduced as indicated and treated with 1 μg·mL^−1^ doxycycline (dox) for 72 h (*n* = 3). (C) Immunofluorescent staining of indicated MS1 cells probed using anti‐FLAG antibody in dox treated cells as in (B), scale bar 50 μm (*n* = 3). (D) Relative expression of TEAD1 transcriptional target *Lats2* or (E) TFE3 transcriptional target *Trpm1* by qRT‐PCR in MS1 cells (*n* = 3). (F) Relative caspase activity of suspension‐cultured MS1 cells normalized to RFP control cells grown in adherent conditions based on Caspase 3/7 Glo Assays (*n* = 3). (G) Focus assay quantification in indicated cells treated with 1 μg·mL^−1^ dox for 72 h post confluence (*n* = 3). (H) Relative expression of indicated mesenchymal or (I) endothelial genes by qRT‐PCR in RFP‐MS1, YT‐MS1, or YT^Δdd^‐MS1 cells treated with dox (1 μg·mL^−1^) (*n* = 3). (J) Relative expression of TFE3 transcriptional target gene, *Trpm1* by qRT‐PCR in 48 h dox (1 μg·mL^−1^) pretreated cells then treated with DMSO or terfenadine (2 μm) for 24 h as indicated, data presented as independent biological replicates normalized to RFP‐DMSO (*n* = 3). (K) Relative Caspase 3/7 activity of suspension‐cultured MS1 cells normalized to RFP control grown in adherent conditions (*n* = 3). (L) Relative expression of indicated mesenchymal (*Acta2* or *Tgm2*) or endothelial (*Epas1* or *Fli1*) genes by qRT‐PCR in MS1 cells treated as in (J), data presented as independent biological replicates normalized to RFP‐DMSO (*n* = 3). Error bars indicate standard deviation; significance *P < 0.05, **P < 0.01, ***P < 0.001, ****P < 0.0001 determined by Student's two‐tailed *t*‐test.

YT retains the DNA‐binding bHLH‐LZ domain, which allows TFE3 to dimerize and bind to CLEAR elements to drive transcription [24]. Previous work has demonstrated that YT is active at TFE3 promoter regions [23, 55]. To determine whether this bHLH‐LZ domain is necessary for YT to drive EndMT, we created a mutant YT with the TFE3‐bHLH‐LZ domain removed to disrupt dimerization and DNA‐binding (Fig. 6A). Similar protein levels were observed for YT and YT^Δdd^ by immunoblot, and immunofluorescence staining revealed that YT^Δdd^ was localized in both the nucleus and cytoplasm (Fig. 6B,C). Nonetheless, increased expression of *Lats2* was observed by qRT‐PCR, suggesting YT^Δdd^ retains the ability to bind TEADs and induce transcription like wildtype YT (Fig. 6D). YT^Δdd^ expression did not recapitulate the increase in expression of TFE3 transcriptional target *Trpm1*, indicating a loss of TFE3‐mediated transcriptional activity (Fig. 6E). Expression of YT^Δdd^ did not confer anoikis resistance, as YT^Δdd^ cells did not exhibit reduced apoptosis in suspension culture conditions compared to control RFP cells (Fig. 6F). Additionally, YT^Δdd^ cells did not form foci in adherent culture after becoming fully confluent (Fig. 6G, Fig. S6A). We determined by qRT‐PCR that YT^Δdd^ cells do not undergo transcriptional changes consistent with EndMT (Fig. 6H,I). These results indicate that the dimerization and DNA‐binding bHLH‐LZ domains of TFE3 are necessary to drive EndMT plasticity.

TFE3's ability to bind to DNA and drive transcription is dependent on dimerization of the bHLH‐LZ domain. Recent research focusing on other TFE3‐rearranged cancers has identified terfenadine, an antihistamine drug, as an inhibitor of dimerization of bHLH‐LZ domains of the Mitf/TFE family of transcription factors [27]. To most closely mimic a clinical treatment, we treated cells with terfenadine postdox induction in YT‐MS1 cells. We determined a very similar IC_50_ for terfenadine in YT‐MS1 cells compared to control (Fig. S6B). Terfenadine treatment of YT‐MS1 cells resulted in a drastic reduction of *Trpm1*, a canonical TFE3 transcriptional target, consistent with a reduction in bHLH‐LZ dimerization and transcriptional activity (Fig. 6J). Terfenadine‐treated YT‐MS1 cells also displayed a significantly higher amount of apoptosis than DMSO‐treated YT cells when cultured in suspension for 24 h, similar to RFP control cells (Fig. 6K). Notably, the RFP‐MS1 control cells are already apoptotic at this time point; thus, we did not include RFP‐MS1 terfenadine‐treated control. Transcriptionally, YT‐MS1 cells lost the robust increase in mesenchymal targets after treatment (Fig. 6L). However, terfenadine was not sufficient to restore the transcriptional levels of endothelial markers decreased with YT expression (Fig. 6L). Taken together, these results suggest targeting dimerization of YT may be effective in partially reversing the EndMT phenotype of EHE cells.

### 3.6 TFE3 overexpression promotes anoikis resistance but does not fully recapitulate YT phenotypes

**Fig. 7:**
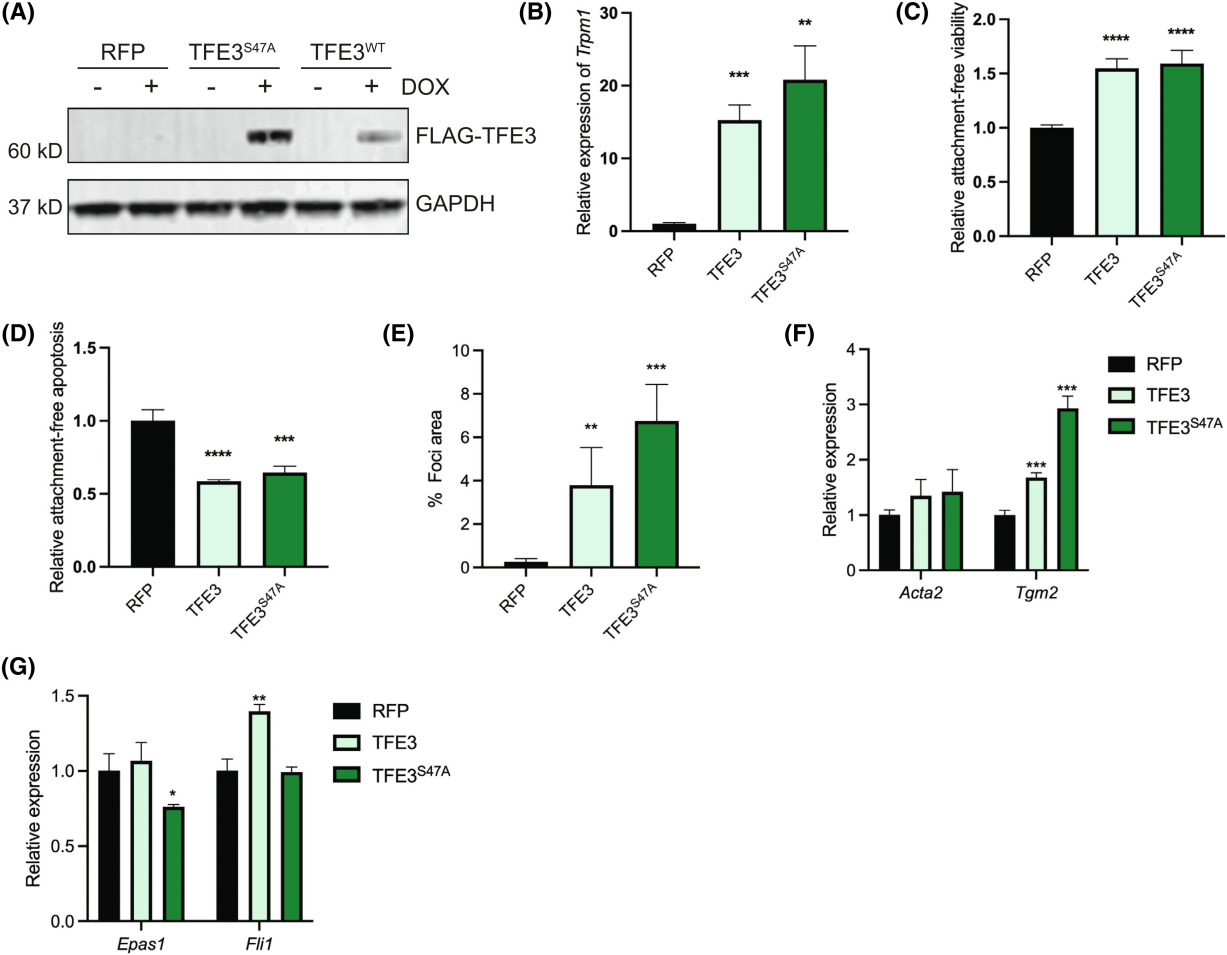
TFE3 overactivity induces endothelial anoikis resistance in MS1 cells. (A) Immunoblot analysis of inducible TFE3 and TFE3^S47A^ in MS1 cells treated ±1 μg·mL^−1^ doxycycline (dox) for 72 h and probed with indicated antibodies (*n* = 3). (B) Relative expression of TFE3 transcriptional target *Trpm1* by qRT‐PCR in RFP‐MS1 (black), TFE3‐MS1 (light green), or TFE3^S47A^‐MS1 (dark green) cells treated with dox for 72 h (1 μg·mL^−1^) (*n* = 3). (C) Relative viability by Cell Titer Glo and (D) relative Caspase activity by Caspase 3/7 Glo assays of suspension‐cultured TFE3‐MS1 and TFE3^S47A^‐MS1 cells normalized to RFP control cells grown in adherent conditions (*n* = 3). (E) Focus formation quantification of RFP‐MS1, TFE3‐MS1, or TFE3^S47A^‐MS1 cells treated with dox (1 μg·mL^−1^) (*n* = 3). (F) Relative expression of indicated mesenchymal or (G) endothelial genes by qRT‐PCR in RFP‐MS1, TFE3‐MS1, or TFE3^S47A^‐MS1 cells treated with 1 μg·mL^−1^ dox (*n* = 3). Error bars indicate standard deviation; significance *P < 0.05, **P < 0.01, ***P < 0.001, ****P < 0.0001 determined by Student's two‐tailed *t*‐test.

We next sought to determine whether TFE3 overexpression and activity are sufficient to drive EndMT and/or transformation in endothelial cells. TFE3 transcriptional activity is low based on undetectable *Trpm1* transcripts by qRT‐PCR in normal MS1 cells. TFE3 is also an unstable protein in nutrient‐rich conditions due to phosphorylation of S47, which activates a CUL1^β‐TrCP^ degron motif to promote its degradation [56]. Thus, we transduced cells with lentiviral dox‐inducible expression of wild‐type TFE3 or a stabilized mutant TFE3 with the phosphorylation site mutated to alanine, TFE3^S47A^ (Fig. 7A). Expression of either wild‐type or TFE3^S47A^ was sufficient to induce expression of the canonical TFE3 transcriptional target *Trpm1* (Fig. 7B). Overexpression of TFE3 is sufficient to drive an anoikis‐resistant phenotype, both reducing apoptosis and increasing viability in suspension cultures compared with control cells (Fig. 7C,D). TFE3 overexpression was unable to recapitulate the formation of large 3D foci driven by YT; however, TFE3 and TFE3^S47A^ expression did cause the formation of small foci that were not observed in control cells (Fig. 7E, Fig. S7A,B). Overexpression of TFE3 or TFE3^S47A^ resulted in upregulation of the mesenchymal gene *Tgm2* but did not drive significant changes in *Acta2* expression. Endothelial marker *Epas1* was slightly but significantly reduced upon TFE3^S47A^ expression, while *Fli1* was slightly increased upon wild‐type TFE3 expression, suggesting no loss of endothelial identity (Fig. 7F,G). These data indicate that TFE3 expression can modulate some phenotypic aspects associated with endothelial transformation but is not sufficient to fully recapitulate the transformation and EndMT effects of YT.

### 3.7 MS1 EHE cells maintain mesenchymal plasticity *in vivo*

**Fig. 8:**
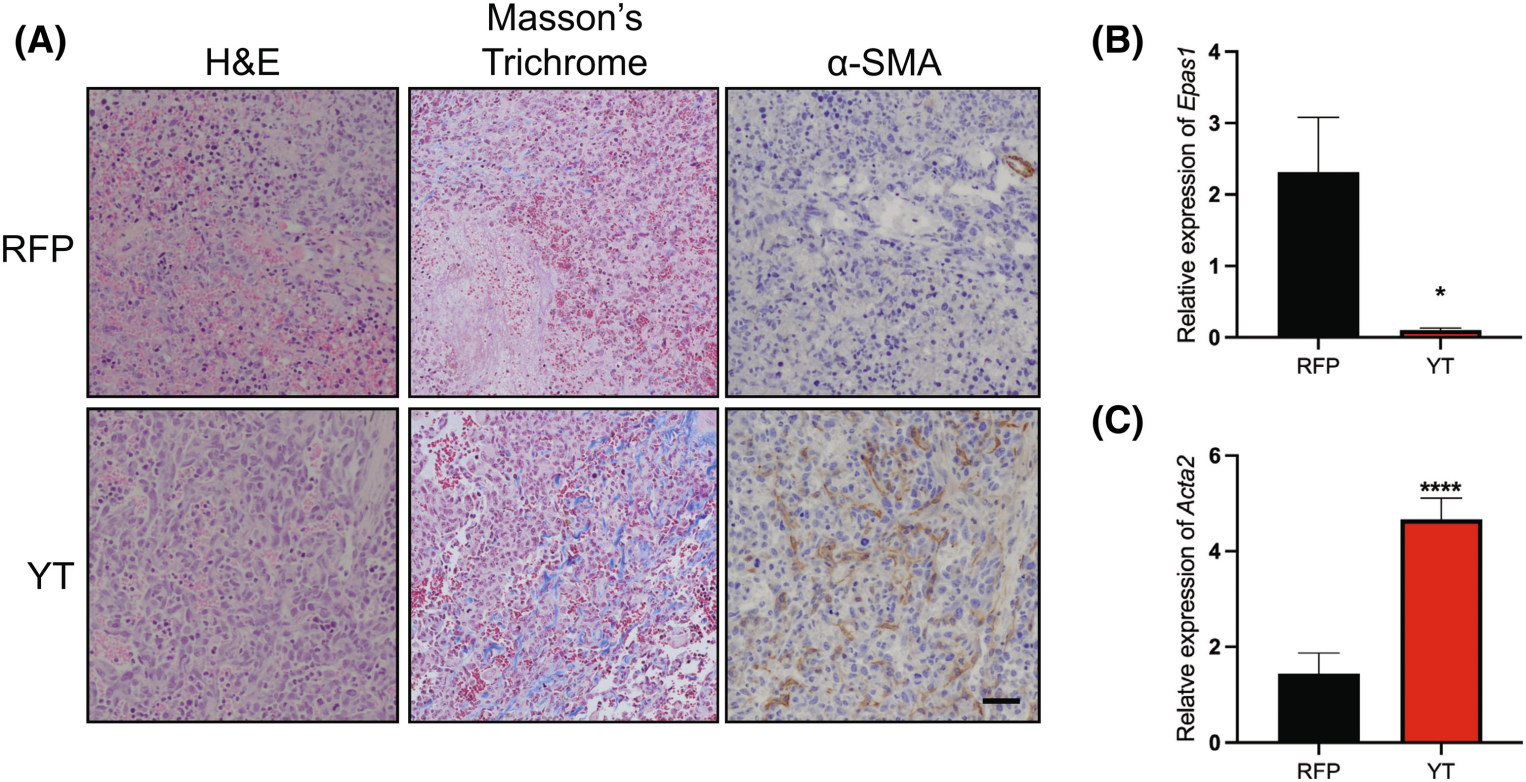
YT‐MS1 cells retain mesenchymal plasticity *in vivo*. (A) Representative hematoxylin and eosin (H&E) staining, Masson's trichrome stain, or ⍺SMA immunohistochemistry (IHC) of indicated RFP‐MS1 control or YT‐MS1 allografts, scale bar 50 μm (*n* = 3). (B) Relative expression of *Epas1* or (C) *Acta2* by qRT‐PCR in RFP‐MS1 (black) or YT‐MS1 (red) allograft tumors (*n* = 3). Error bars indicate standard deviation; significance *P < 0.05, ****P < 0.0001 determined by Student's two‐tailed *t*‐test.

The transcriptional profile of endothelial cells is sensitive to external cues, such as shear stress and cell culture conditions [57]. When injected subcutaneously into immunodeficient mice, MS1 cells form slow‐growing hemangiomas [49]. To determine whether YT alters tumor growth and EndMT phenotypes *in vivo*, we generated allograft tumors by injecting the YT‐MS1 or ‐RFP control cells subcutaneously into NRG mice. Although tumor growth kinetics were not significantly different between YT‐expressing and control cells, striking pathologic differences were observed. RFP tumors exhibited significant areas of necrosis suggesting impaired vascularization, whereas YT tumors displayed more uniformly viable and vasoformative tumors (Fig. 8A). YT allografts displayed significant collagen depositions surrounding the neoplastic cells throughout the tumors based on Masson's trichrome staining. These collagen and ECM elements indicate an active remodeling of the tumor stroma consistent with EndMT. While it is possible that this collagen is produced from activated fibroblasts, which can also be attributed to EndMT, YT expression significantly increased the transcript level of several collagen genes based on RNA‐seq analysis of cultured YT‐MS1 cells (Fig. S8). Using immunohistochemistry, we observed strong ⍺‐SMA (ACTA2) staining in YT allografts, whereas ⍺‐SMA staining in RFP allografts was restricted to perivascular cells and the tumor periphery (Fig. 8A). Finally, qRT‐PCR of RNA isolated from allografts revealed transcriptional upregulation of *Acta2* and loss of endothelial *Epas1* expression (Fig. 8B,C).

## 4 Discussion

Malignant vascular sarcomas such as EHE and angiosarcoma (AS) represent a subset of deadly rare diseases fraught with hurdles for scientific research. Limited models exist, particularly patient‐derived models, due to low biospecimen availability and challenges in culturing primary endothelial cells due to sensitivities to cell culture conditions [48, 58]. Therefore, there is a strong interest in the generation and characterization of genetically engineered cell lines and mouse models [46, 47]. Vascular sarcomas are diverse in both clinical presentation and mutational genomic landscape and will require a diverse range of preclinical models to tease apart the underlying dependencies.

To better understand the molecular drivers and dependencies driving EHE pathogenesis, we engineered endothelial EHE cell models using murine and human endothelial cell lines. In line with previous studies [23], we found that TC expression alone did not drive a robust phenotype comparable to YT cells. Recent studies have shown TC induces transcriptional stress and senescence in endothelial cells [51]. Additionally, many patient tumors harboring TC exhibit co‐occurring alterations, such as *CDKN2A* deletion, which may be essential for enhancing TC‐driven transformation in endothelial cells [52]. However, we found that YT is sufficient to transform cells, which undergo significant morphological changes and lose contact inhibition.

Transcriptomic analysis of YT‐expressing MS1 cells revealed enrichment of EHE‐associated genes. Gene ontology analysis indicated considerable changes in the expression of genes involved in cell adhesion, ECM organization, and migration. We observed a downregulation of genes associated with both “vasculature development” and “ECM organization,” reflecting the loss of endothelial characteristics associated with the endothelial‐to‐mesenchymal transition (EndMT).

During development, endothelial progenitors undergo EndMT, where cells gain expression of genes associated with mesenchymal cells and lose expression of endothelial genes [1]. EndMT is an essential developmental process, and recent studies suggest that a partial EndMT state has an important role in sprouting angiogenesis, promoting the formation of highly migratory tip cells [4]. EndMT promotes migratory phenotypes through loss of cell–cell junctions, cytoskeletal changes, and resistance to anoikis. Erroneous EndMT activation is associated with pathophysiologic functions, such as atherosclerosis [4, 59, 60], pulmonary hypertension [1, 61, 62], vascular malformations [5, 6], and cancer [2, 3, 4]. EndMT has also been found to support various therapeutic resistance mechanisms, including chemotherapy [2, 3, 62], radiotherapy [2, 3], and antiangiogenic therapy [63]. To investigate the EndMT gene signature identified by GO analysis, we evaluated such EndMT‐associated phenotypes with our EHE model cells. We confirmed that YT‐EHE cells had an increased migratory ability and a striking resistance to the anoikis cell‐death pathway.

As EHE fusions TC and YT contain the TEAD‐binding domains (TBD) of the paralogs TAZ and YAP1, respectively, EHE has been considered a cancer primarily driven through unrestrained Hippo pathway activity [18, 19, 20, 22, 23]. The Hippo pathway is considered the master controller of organ and tissue growth in animals [14, 17, 64], of which TAZ and YAP1 are the major downstream effectors.

To better understand the relationship between TEAD activity and the observed EndMT phenotypes, we generated a mutant YT^S94A^ with a nonfunctional TBD. YT^S94A^ cells maintained an EndMT phenotype similar to that of YT cells, if less pronounced. These results were confirmed using K‐975, a TEAD inhibitor [54]. Taken together, these results suggest that TEAD transcription contributes meaningfully to transformative phenotypes in YT‐EHE cells but is not required for EndMT plasticity. This surprising finding may be unique to YT fused EHE tumors; previous evidence suggests TC expressing cells are sensitive to TEAD inhibition [23, 52].

The TFE3‐bHLH‐LZ domain is conserved in the YT fusion protein at all described break points. TFE3 is known to be fused with other N‐terminal partners from chromosomal translocation events, resulting in functional proteins, which promote multiple tumor types [25, 28, 29, 30, 65]. Of particular interest are TFE3 fusions, which drive translocation‐positive renal cell carcinoma (tRCC), some of which have been shown to drive epithelial to mesenchymal transition (EMT) in renal epithelial cells [26]. Previous studies have demonstrated that YT maintains the ability to activate TFE3 promoter regions [23, 55]. YT lacking the bHLH‐LZ domain did not recapitulate the EndMT phenotype driven by expression of YT. These results suggest that the TFE3‐bHLH‐LZ mediated activities are necessary for driving transformation and EndMT.

Recent research focusing on other TFE3‐rearranged cancers has identified terfenadine, an antihistamine drug, as an inhibitor of dimerization of the bHLH‐LZ domain of TFE3 [27]. We found that treating with terfenadine after doxycycline induction of YT expression effectively reduced the transcription of mesenchymal genes down to near‐baseline levels. However, it did not restore the expression of endothelial genes, perhaps suggesting chromatin modifications at endothelial genes may be responsible for reduced transcription. Terfenadine treatment was effective in preventing anoikis resistance and resulted in a reduced mesenchymal state, restricting the plasticity of the cells. Although terfenadine is unsuitable for clinical use due to toxicities, interest in discovering safe TFE family dimerization inhibitors for cancer therapy remains high [27]. These results represent further proof of concept for the potential of TFE3 dimerization inhibitors as therapeutics for improving YT‐EHE patient outcomes. These inhibitors could be used alone or in combination with other small molecules, such as MEK or mTOR inhibitors, currently being tested in EHE patients [66, 67].

Overexpression of TFE3 did not lead to consistent transcriptional changes in line with EndMT. However, MS1 cells expressing either TFE3 or TFE3^S47A^ exhibited anoikis resistance and a subtle promotion of foci formation. Therefore, YT promotes oncogenic activities beyond TFE3 overexpression, and TFE3 overexpression alone is not sufficient to cause EndMT in MS1 cells. Notably, inhibition of TFE3 activity was able to suppress most of the phenotypic effects of YT, highlighting the necessity of TFE3 function. This discrepancy, where TFE3 activity is necessary but not sufficient, could be accounted for by several potential mechanisms. The YAP1 domains within the fusion protein may enhance the transcriptional activity of YT through TEAD‐dependent and/or TEAD‐independent mechanisms. Additionally, YAP1 domains may facilitate the recruitment of interacting proteins not activated or engaged by TFE3 alone, leading to enhanced transcriptional activity or alteration in the chromatin landscape. These potential mechanisms warrant further investigation.

## 5 Conclusions

In this study, we characterized the phenotypic and transcriptional changes in endothelial cells caused by the expression of EHE fusion proteins. Surprisingly, we found that the expression of fusion proteins TC and YT induced distinct phenotypes, and that YT in particular was able to drive EndMT. Due to the ability of YT to activate TFE3‐responsive promoters, it may share molecular dependencies with other rare TFE3‐rearranged tumors, such as tRCC and perivascular epithelioid cell tumors, which have overlapping features [65, 68]. A deeper understanding of the molecular similarities between YT‐EHE and other TFE3‐rearranged tumors may ultimately drive the development of new therapeutic strategies and improved clinical outcomes for patients.

## Conflict of interest

The authors declare no conflict of interest.

## Author contributions

JH and AM conceived the project and designed experiments. AM, SH, PVC, SJ, MY, NJ, BL, AB, MK, and JH performed experiments, collected data, analyzed, and visualized the data. AM and JH wrote the original draft. AM, SH, PVC, SJ, MY, NJ, BL, AB, MK, and JH reviewed and edited the manuscript. JH acquired funding. AM, MK, and JH supervised the studies. All authors read, reviewed, and approved the manuscript.

## Supporting information


**Fig. S1.** Generation of human endothelial EHE cell models.
**Fig. S2.** YT‐HUVECs have altered morphology.
**Fig. S3.** Altered gene expression in YT‐MS1 cells.
**Fig. S4.** YT‐expressing cells demonstrate an EndMT transcriptional signature.
**Fig. S5.** TEAD activity is dispensable for loss of contact inhibition in YT cells.
**Fig. S6.** DNA binding and dimerization bHLH‐LZ domains of YT are necessary for loss of contact inhibition growth in YT‐MS1 cells.
**Fig. S7.** TFE3 overexpression does not fully recapitulate YT‐driven EndMT phenotypes.
**Fig. S8.** Differential expression of collagen genes with YT expression.

**Table S1.** SYBR primers used for qRT‐PCR.
**Table S2.** Primers for molecular cloning and mutagenesis.
**Table S3.** Antibodies used for immunoblots, immunoprecipitations, immunohistochemistry (IHC), and immunofluorescence (IF).
**Table S4.** Gene Ontology analysis of downregulated genes in MS1 cells expressing YT.
**Table S5.** Gene Ontology analysis of upregulated genes in MS1 cells expressing YT.

## Data Availability

The RNA‐sequencing transcriptomic data are deposited in the Gene Expression Omnibus under accession number GSE287795.
