# Supplementary material for: YAP1::TFE3 mediates endothelial‐to‐mesenchymal plasticity in epithelioid hemangioendothelioma
Source: Mol Oncol. 2025 Aug 17;20(2):493–510. doi: 10.1002/1878-0261.70112 (PMC12936420; doi:10.1002/1878-0261.70112)
Supplement: Supplementary file 1 — Fig. S1. Generation of human endothelial EHE cell models. Fig. S2. YT‐HUVECs have altered morphology. Fig. S3. Altered gene expression in YT‐MS1 cells. Fig. S4. YT‐expressing cells demonstrate an EndMT transcriptional signature. Fig. S5. TEAD activity is dispensable for loss of contact inhibition in YT cells. Fig. S6. DNA binding and dimerization bHLH‐LZ domains of YT are necessary for loss of contact inhibition growth in YT‐MS1 cells. Fig. S7. TFE3 overexpression does not fully recapitulate YT‐driven EndMT phenotypes. Fig. S8. Differential expression of collagen genes with YT expression. [file MOL2-20-493-s002.pdf]

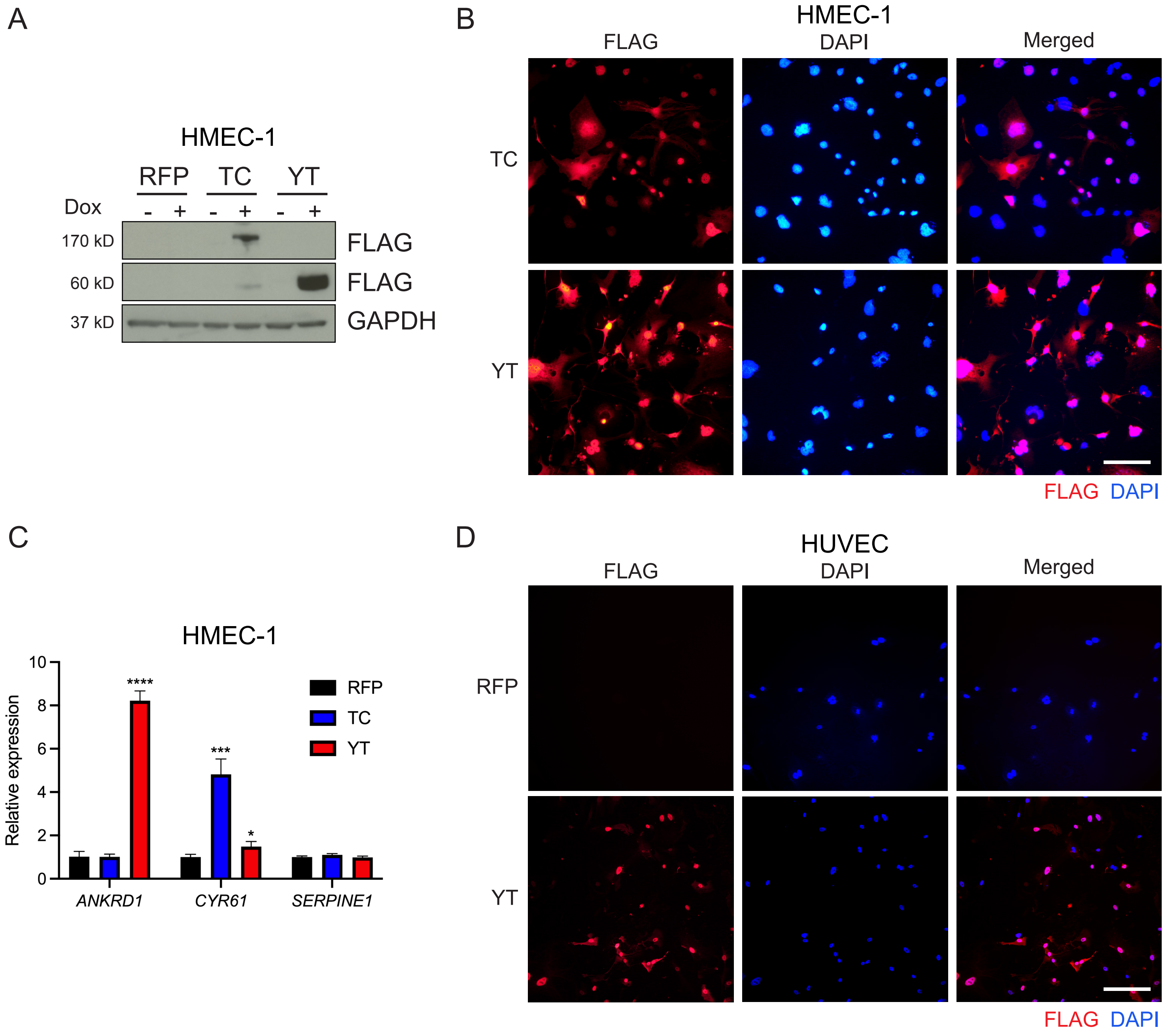

Supplemental Figure S1. Generation of human endothelial EHE cell models. (A) Immunoblot analysis of HMEC-1 cell lysates 72 hours post +/-1 µg/mL doxycycline (dox) treatment and probed with anti-FLAG or GAPDH as indicated (*n* = 3). **(B)** Immunofluorescent staining of indicated HMEC-1 cells using anti-FLAG antibody in dox treated cells as in (A), scale bar 50 µm. **(C)** Relative expression of indicated canonical Hippo pathway regulated genes by qRT-PCR in HMEC-1 cells as in (A) (*n* = 3). **(D)** Immunofluorescent staining of indicated HUVEC cells probed using anti-FLAG antibody in dox treated cells as in (A), scale bar 50 µm (*n* = 3). Error bars indicate standard deviation, significance *p<0.05, ***p<0.001, ****p<0.0001 determined by Student's two-tailed *t*-test.

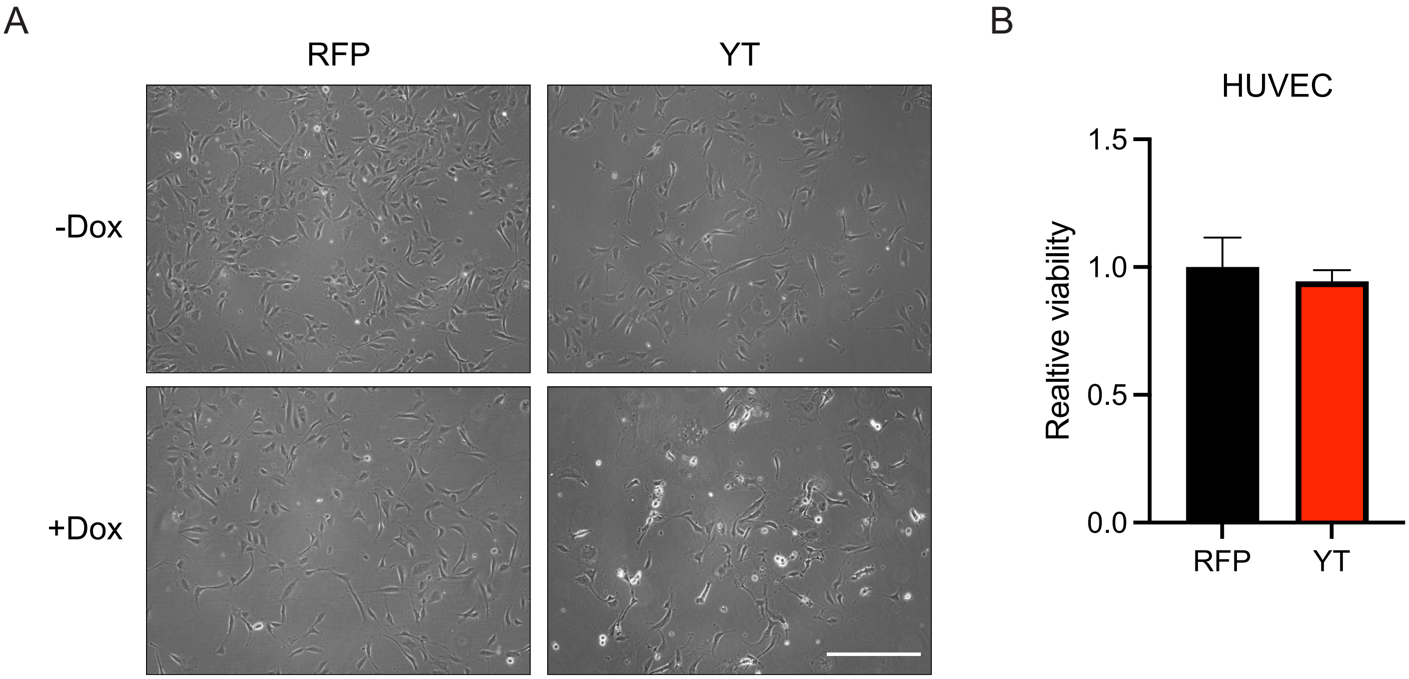

Supplemental Figure S2. YT-HUVEC cells have altered morphology. (A) Brightfield images of HUVEC-pCW-RFP control or pCW-YT cells 72 hours post +/- 1μg/mL doxycycline treatment, scale bar 100 μm (*n* = 3). **(B)** Relative cell viability of HUVEC cells as in (A) based on Cell Titer Glo (*n* = 3). Error bars indicate standard deviation, significance determined by Student's two-tailed *t*-test.

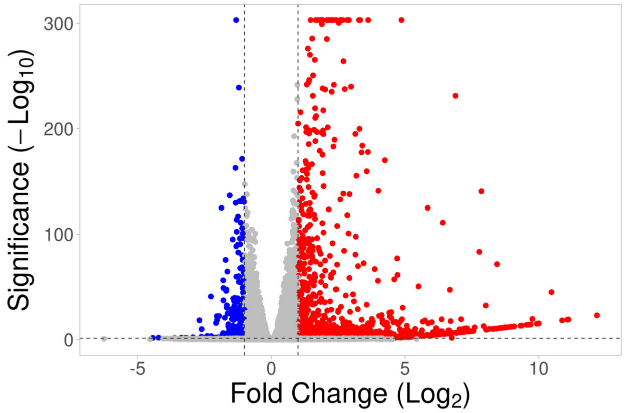

Supplemental Figure S3. Altered gene expression in YT-MS1 cells. Volcano plot of the $-\log_{10}$ of the p value versus the $\log_2$ fold change in gene expression of RFP compared to YT expressing MS1 cells treated with dox (1 μg/mL) for 72 hours, indicated upregulated (red) and downregulated (blue) genes with $\log_2$ fold change >1 and <-1, respectively with $p < 0.05$.

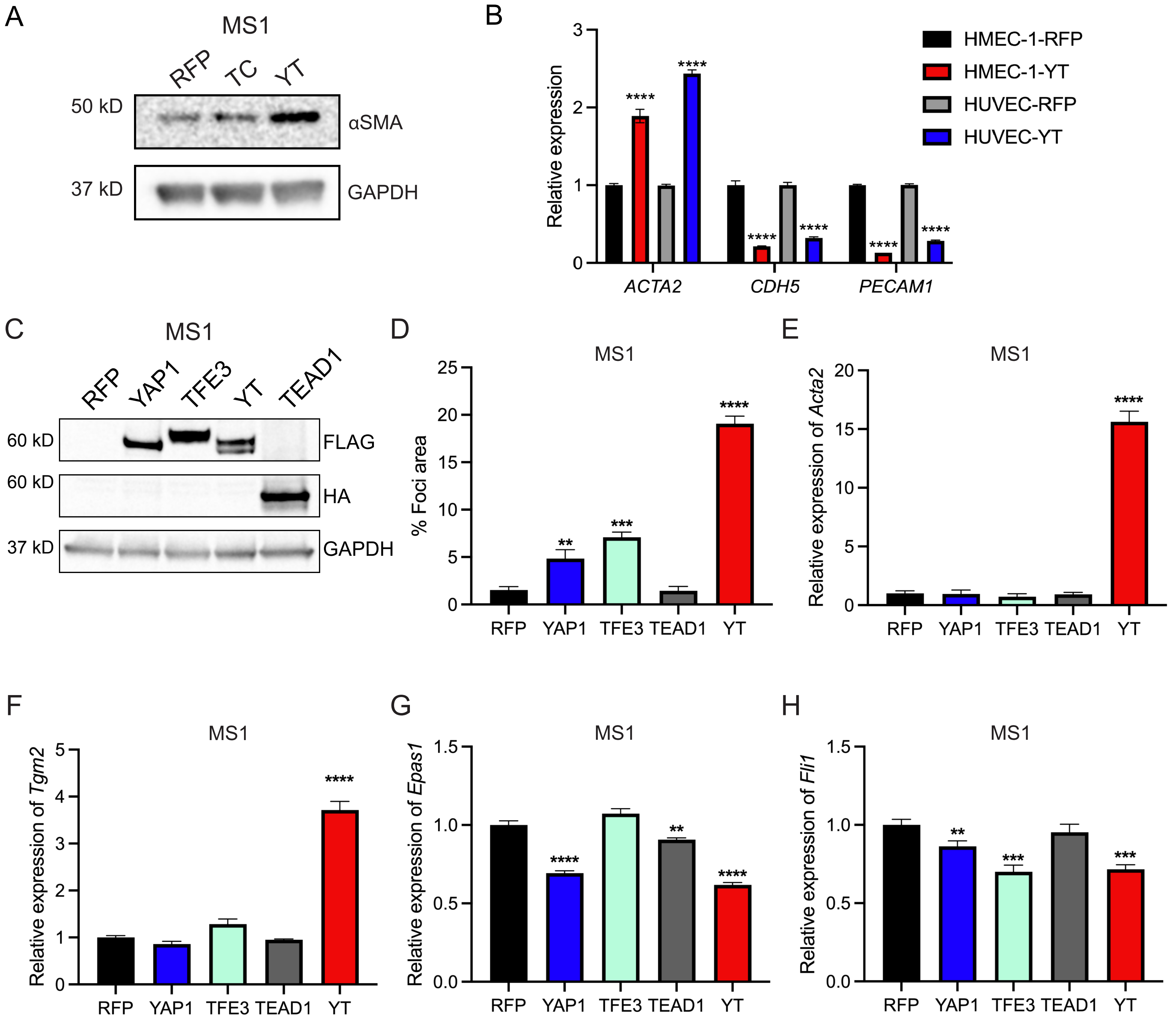

Supplemental Figure S4. YT-expressing cells demonstrate an EndMT transcriptional signature. (A) Immunoblot analysis of lysates from indicated MS1 cells treated with 1 μg/mL doxycycline (dox) for 72 hours (*n* = 3). **(B)** Relative expression by qRT-CR of canonical EndMT genes in indicated human endothelial cells treated with dox as in (A) (*n* = 3). **(C)** Immunoblot analysis of lysates from indicated MS1 cell lysates treated with dox as in (A) and probed with anti-FLAG, HA, or GAPDH (*n* = 3). **(D)** Quantification of foci formation assays in indicated MS1 cells treated as in (A) (*n* = 3). **(E)** Relative expression by qRT-PCR in MS1 cells as in (D) for indicated EndMT genes including *Acta2*, **(F)** *Tgm2*, **(G)** *Epas1*, or **(H)** *Fli1* (*n* = 3). Error bars indicate standard deviation, significance **p<0.01, ***p<0.001, ****p<0.0001 determined by Student's two-tailed *t*-test.

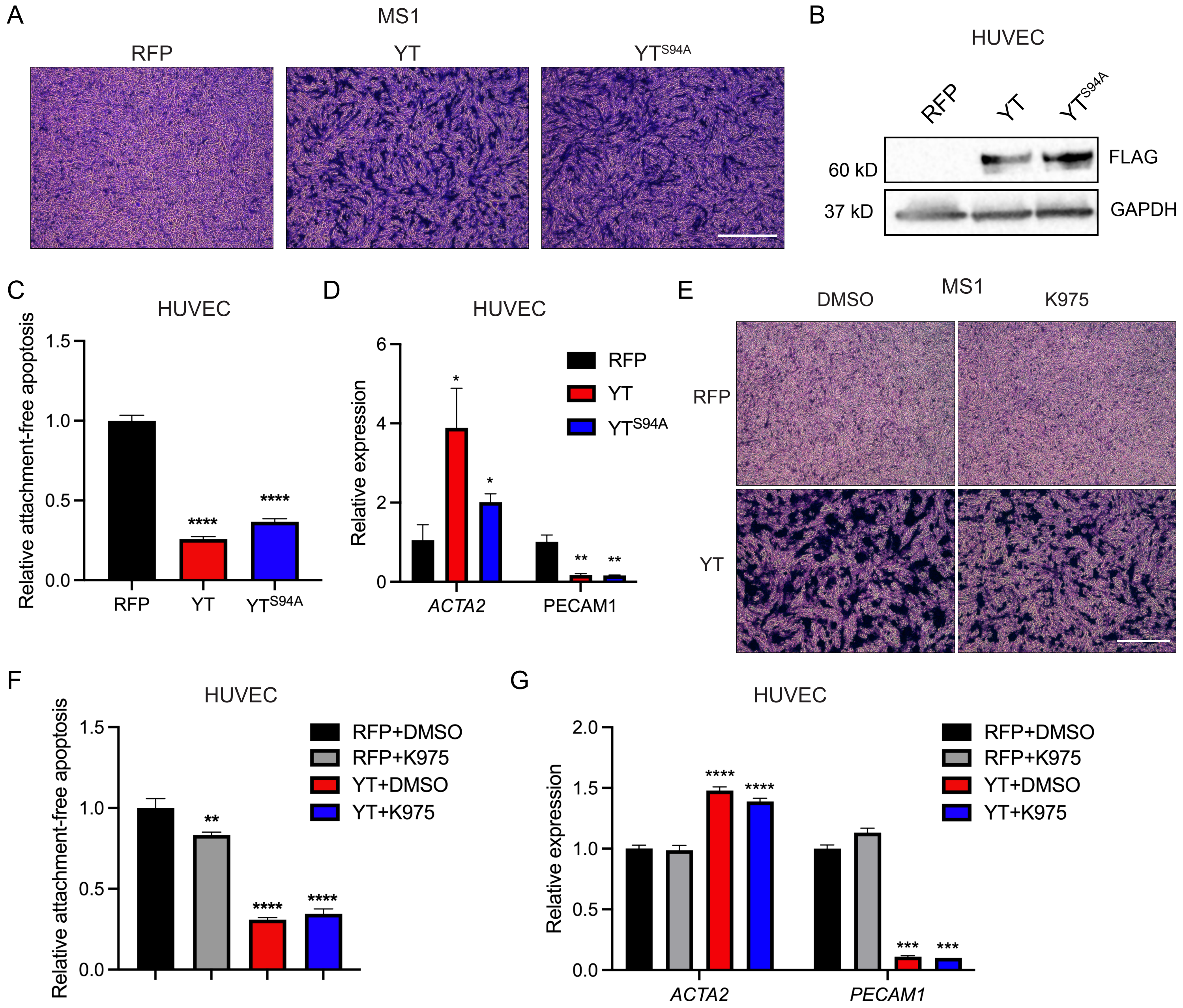

Supplemental Figure S5. TEAD activity is dispensable for loss of contact inhibition in YT cells. (A) Representative images of crystal violet stained focus assay of indicated MS1 cell line treated with 1 µg/mL doxycycline (dox), scale bar 750 µm (*n* = 3). **(B)** Immunoblot of HUVEC-RFP, -YT, or -YTS94A treated with 1 µg/mL dox for 72 hours and probed with anti-FLAG or GAPDH as indicated (*n* = 3). **(C)** Relative Caspase activity of dox treated suspension cultured HUVEC cells normalized to RFP control cells and cells grown in adherent conditions (*n* = 3). **(D)** Relative expression of *ACTA2* or *PECAM1* by qRT-PCR of indicated HUVEC cells treated with dox for 72 hours (*n* = 3). **(E)** Representative focus assay images of RFP-MS1 control or YT-MS1 cells treated with 1 µg/mL dox and 1 µM K-975 or DMSO for 72 hours (*n* = 3), scale bar 750 µm. **(F)** Relative Caspase activity of indicated HUVEC cells cultured in suspension and treated with dox and DMSO or 1µM K-975 for 72 hours. Data normalized to RFP control cells and cells grown in adherent conditions (*n* = 3). **(G)** Relative expression of *ACTA2* or *PECAM1* by qRT-PCR of indicated HUVEC cells treated with dox and DMSO or K-975 as indicated for 72 hours (*n* = 3). Error bars indicate standard deviation, significance *p<0.05, **p<0.01, ***p<0.001, ****p<0.0001 determined by Student's two-tailed *t*-test.

A

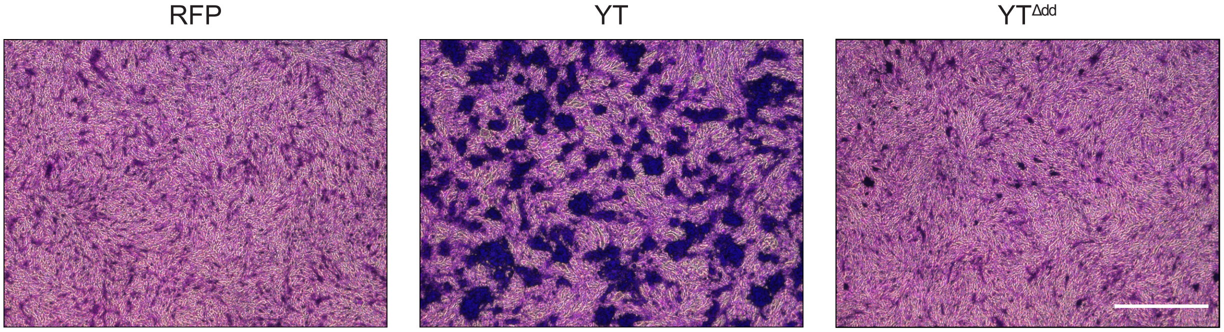

B

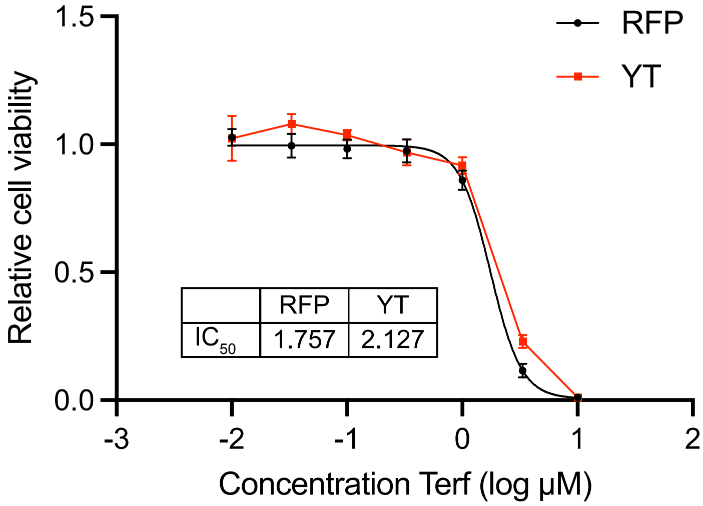

Supplemental Figure S6. DNA binding and dimerization bHLH-LZ domains of YT are necessary for loss of contact inhibition growth in YT-MS1 cells. (A) Representative images of focus assays in indicated MS1 cell line after 72 hours with 1 µg/mL doxycycline (dox) treatment, scale bar 750 µm (*n* = 3). **(B)** IC_{50} cell viability curves for dox treated RFP-MS1 (black) or YT-MS1 cells (red), 72 hours after treatment with dox and indicated concentrations of bHLH-LZ dimerization inhibitor, terfenadine. Error bars indicate standard deviation.

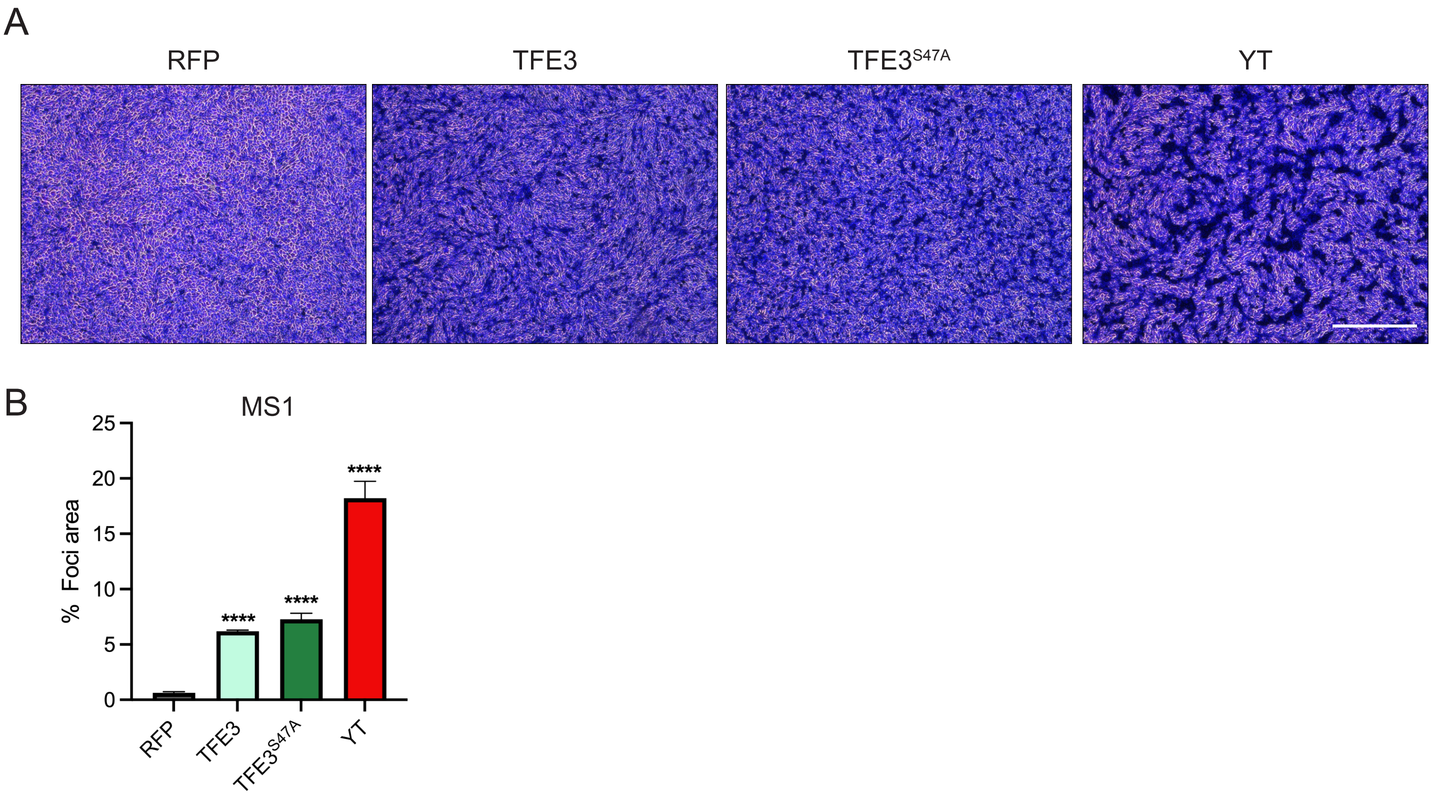

Supplemental Figure S7. TFE3 overexpression does not fully recapitulate YT-driven EndMT phenotypes. (A) Representative images of crystal violet stained focus assays from indicated MS1 cell lines treated with 1 μg/mL doxycycline, scale bar 750 μm (*n* = 3). **(B)** Focus formation quantification of cells from (A) (*n* = 3). Error bars indicate standard deviation, significance ****$p<0.0001$ determined by Student's two-tailed *t*-test.

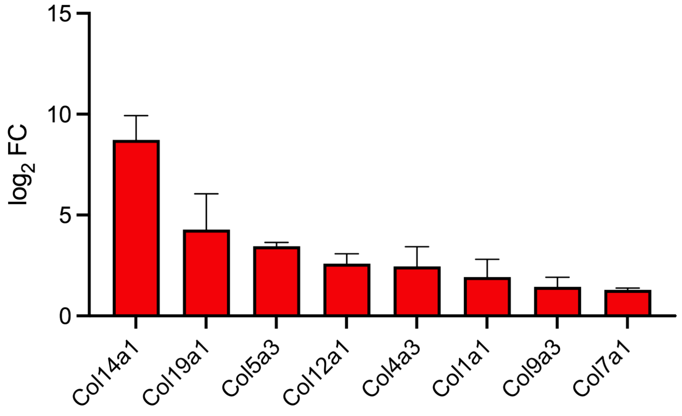

Supplemental Figure S8. Differential expression of Collagen genes with YT expression. All collagen genes with increased expression based on $\log_2$ fold change (FC) > 1 and $p < 0.05$ in YT-MS1 cells compared to RFP-MS1 cells from RNA-seq analysis as in Figure 3. Error bars indicate the standard error of the $\log_2$ fold change.
